# In-plane anisotropic electronics based on low-symmetry 2D materials: progress and prospects

**DOI:** 10.1039/c9na00623k

**Published:** 2019-12-06

**Authors:** Siwen Zhao, Baojuan Dong, Huide Wang, Hanwen Wang, Yupeng Zhang, Zheng Vitto Han, Han Zhang

**Affiliations:** International Collaborative Laboratory of 2D Materials for Optoelectronics Science Technology of Ministry of Education, Key Laboratory of Optoelectronic Devices and Systems of Ministry of Education and Guangdong Province, Shenzhen University Shenzhen 518060 China hzhang@szu.edu.cn; Shenyang National Laboratory for Materials Science, Institute of Metal Research, Chinese Academy of Sciences Shenyang 110000 China vitto.han@gmail.com; School of Material Science and Engineering, University of Science and Technology of China Anhui 230026 China

## Abstract

Low-symmetry layered materials such as black phosphorus (BP) have been revived recently due to their high intrinsic mobility and in-plane anisotropic properties, which can be used in anisotropic electronic and optoelectronic devices. Since the anisotropic properties have a close relationship with their anisotropic structural characters, especially for materials with low-symmetry, exploring new low-symmetry layered materials and investigating their anisotropic properties have inspired numerous research efforts. In this paper, we review the recent experimental progresses on low-symmetry layered materials and their corresponding anisotropic electrical transport, magneto-transport, optoelectronic, thermoelectric, ferroelectric, and piezoelectric properties. The boom of new low-symmetry layered materials with high anisotropy could open up considerable possibilities for next-generation anisotropic multifunctional electronic devices.

## Abstract 2

Low-symmetry layered materials such as black phosphorus (BP) have been revived recently due to their high intrinsic mobility and in-plane anisotropic properties, which can be used in anisotropic electronic and optoelectronic devices.

## Graphical abstract

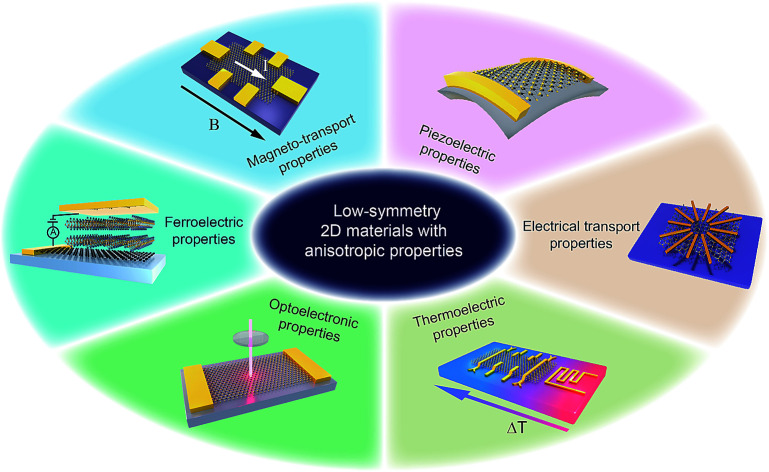

## 1. Introduction

Two dimensional (2D) layered materials with strong in-plane covalent bonds and weak out-of-plane van der Waals interactions span a very broad range of solids and exhibit extraordinary and unique layer-dependent physical properties after the discovery of graphene.^1–6^ Even though graphene has extremely large mobility and outstanding electron-transport properties, the absence of a band gap restricts its applications in (opto)electronic devices. Beyond graphene, 2D layered materials have become more and more popular among researchers due to their unique structural,^7,8^ mechanical,^9^ electrical,^10,11^ thermoelectric,^12–15^ optical,^16–22^ catalytic,^23,24^ and sensing properties,.^25–30^ Transition metal dichalcogenides (TMDCs) with tunable band gap fully exert the advantages in low-cost, flexible, and high-performance logic and optoelectronic devices, such as field-effect transistors (FETs), photodetectors, photonic devices and solar cells. However, people mainly focus on the in-plane isotropic behaviors in graphene and TMDCs because of their symmetric crystal structures until the rediscovery of low-symmetry black phosphorus (BP).

It is known that reducing the symmetry of materials is generally associated with exceptional anisotropy in electronic energy band structure and can be regarded as a process of lowering the dimensionality of the carrier transport. Therefore, the electrical, optical, thermal, and phonon properties of these anisotropic materials are diverse along the different in-plane crystal directions. Since these unique intrinsic angle-dependent properties of low-symmetry 2D materials cannot be easily realized in highly symmetric 2D materials, the emergence of in-plane anisotropic properties can provide another new degree of freedom to tune the previous unexplored properties and supply a tremendous opportunity to the design of new devices, such as polarization sensitive photodetectors,^31,32^ polarization sensors,^33^ artificial synaptic devices,^34^ digital inverters,^35^ and anisotropic memorizers^36^ that are highly desired in integrated logic circuits. Thus, BP and other low-symmetry layered materials (Table 1) have attracted enormous research interest towards potential applications and become a hot topic in the community of nanoscience and nanotechnology.

**Table 1:** Low-symmetry 2D layered materials classified by the crystal structure and space group and their basic parameters

Crystal system	Space group (bulk)	Materials	Band gap	Absorption coefficient	Band structure	Effective mass along different directions	Mobility ratio (*μ*_max_/*μ*_min_)	Anisotropic conductance	Ref.
Orthorhombic	*Cmca*	BP	0.35 eV	10^4^ to 10^5^ cm^−1^ (visible region)	Direct	Hole: 6.35/0.15	1.5	1.5	22
						Electron: 1.12/0.17			
	*Pnma*	SnS	1.3 eV	10^5^ cm^−1^ (visible region)	Indirect	Hole: 0.21/0.36	≈1.7	∼2.0	23
		SnSe	0.86 eV	>10^5^ cm^−1^ (visible region)	Indirect	Electron: 0.14/0.08	is ∼5.8	∼3.9	24
		GeS	∼1.55–1.65 eV	1.2 × 10^5^ cm^−1^ (2.0 eV)	Indirect (1,2 L) to direct (3 L)				41
		GeSe	1.1–1.2 eV	10^5^ cm^−1^ (visible region)	Indirect	Hole: 0.33/0.16	1.85	≈3	42
		Sb_2_Se_3_	1.03 eV	>10^5^ cm^−1^ (visible region)	Direct				43
	*Cmc*2_1_	SiP	1.69 to 2.59 eV		Direct (monolayer) to indirect (multilayer)				44
	*Pbam*	GeAs_2_	0.98 eV		Indirect	Hole: 0.65 : 0.41	Hole ≈ 1.9	1.8	45
						Electron: 0.57 : 0.14			
	*Pmn*2_1_	1T′ MoS_2_						1.8	46
		Td-MoTe_2_			Type II Weyl semimetal				47
		Td-WTe_2_			Type II Weyl semimetal	1.64			48
		TaIrTe_4_			Type II Weyl semimetal		1.8–2.2 (10–100 K)	1.7 (300 K)	49
	*Cmcm*	Ta_2_NiS_5_	0.2 eV		Direct	Electron: 3.64/0.34		1.41	50
						Hole: 0.79/0.39			
		ZrTe_5_			Dirac semimetal			1.5	51
Monoclinic	*C*2/*m*	GeP	1.68 eV for monolayer to 0.51 eV for bulk		Indirect	Hole: 0.98/0.57		1.52	52
						Electron: 0.72/0.4			
		GeAs	0.57 (bulk) to 1.66 eV (monolayer)		Indirect		4.6		53
		GaTe	∼1.7 eV	>10^4^ cm^−1^ (visible region)	Direct	∼10		1000 at *V*_g_ = −40V	36
	*P*2_1_/m	TiS_3_	0.8–1 eV		Direct		2.3	4.4	54
		ZrS_3_	2.56 eV		Direct				55
	*P*2_1_/*c*	GeS_2_	3.71 eV	≈1.37 × 10^4^ cm^−1^ (4 eV)	Indirect				
		β-GeSe_2_	2.74 eV	10^4^ cm^−1^	Direct	Hole: 1.562/0.755	2.1	1.58	56
		MoO_2_			Metallic			10.1	57
Triclinic	*P*11̄	ReS_2_	1.35 eV		Direct		3.1	7.5	35
		ReSe_2_	1.2–1.3 eV		Indirect				58
		MP_15_ (M = Li, Na, K)	1.16–1.52 eV		Indirect	Hole: 1.39–2.7	10–100		59
						Electron: 4.44–24.75			
Trigonal	*P*3_1_21	α-Te	∼0.35 eV in bulk and ∼1 eV in monolayer	<1.6 μm is 4.5 × 10^6^ cm^−1^	Indirect along Te chains and direct perpendicular to Te chain	0.32/0.3	≈1.43	1.35	60
Tetragonal	*I*4/*mcm*	TlSe	0.73 eV		Indirect	Hole: 0.64 : 0.35			61

Moreover, strong in-plane anisotropic transport properties of low-symmetry 2D materials are typically a result of the different energy band structure along the different in-plane directions of the layered crystal lattice, leading to drastically different carrier effective mass along the different crystal directions. Therefore, the study on anisotropic magneto-transport properties of low-symmetry layered 2D materials could offer a powerful and useful tool to investigate energy band structures and new physical phenomena of low-symmetry layered 2D materials, such as anisotropic weak localization, anisotropic superconducting, and anisotropic non-linear magneto-resistance, which provide more a comprehensive understanding of their physical properties and insights into potential applications.

In addition, due to the anisotropy of transport properties offered by low-symmetry layered 2D materials, their optoelectronic, thermoelectric, piezoelectric, and ferroelectric properties should also be dependent on the crystalline directions. There is no doubt that the corresponding performance along a certain crystalline direction is better than the others. Therefore, investigating the anisotropic electronic properties along different crystalline orientations in low-symmetry 2D materials can optimize the performance of field effect transistors,^35^ photodetectors,^36^ thermoelectric devices,^15^ piezoelectric devices,^37^ ferroelectric devices,^38^ and so on. Some anisotropic semimetals exhibit large non-saturating magnetoresistance (MR) along a specular orientation and can be used in magnetic devices, *e.g.*, magnetic sensors and magnetic memories.^37–40^ Therefore, the study on anisotropic electronic properties in low-symmetry 2D materials is of considerable interest and importance.

**Fig. 1:**
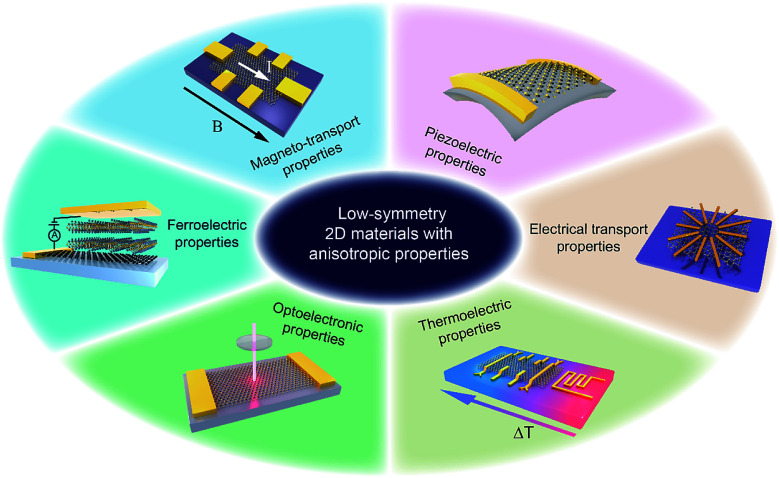
Low-symmetry 2D layered materials with anisotropic electrical transport, magneto-transport, optoelectronic, thermoelectric, piezoelectric, and ferroelectric properties.

Herein, we summarize the recent advances in low-symmetry layered materials and their anisotropic electrical properties. We firstly classify these low-symmetry layered materials by the periodic table of elements and crystal structures. Secondly, we introduce the synthetics methods and their relative merits of these materials, followed by the common methods for characterizing the anisotropy including polarization-dependent absorption spectroscopy (PDAS), azimuth-dependent reflectance difference microscopy (ADRDM), angle-resolved polarization Raman spectroscopy (ARPRS), and angle-resolved DC conductance. Then, the anisotropic electronic properties, *e.g.*, optoelectronic, magneto-transport, thermoelectric, piezoelectric, and ferroelectric properties (Fig. 1) with the applications using them are introduced and discussed. In the end, we conclude the challenges encountered and the future prospects of low symmetry layered materials.

## 2. Crystal structure and electronic band structure

**Fig. 2:**
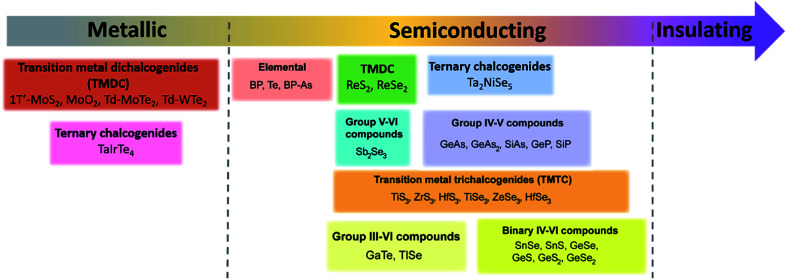
The categorized low-symmetry 2D layered materials by the conductivity and periodic table of elements.

Since materials' anisotropic properties and functionalities are strongly related to their crystal structures and compositions, it is crucial to study the low-symmetry 2D layered crystal structures first. After early investigations on the characterization of structures and properties of bulk samples, the family of low-symmetry 2D layered materials have recently attracted tremendous attentions due to the novel anisotropic properties. Here, we will categorize the low-symmetry 2D layered materials through the conductivity and periodic table of elements as shown in Fig. 2.

### 2.1 Elementary 2D material

**Fig. 3:**
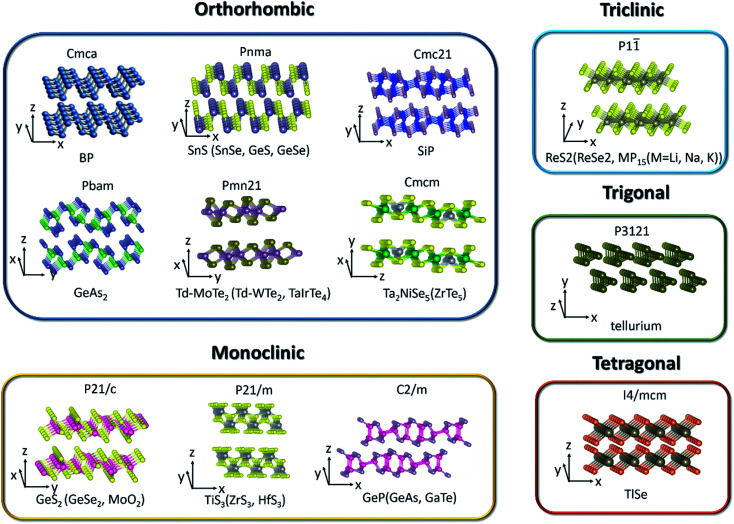
The crystal structure of low-symmetry 2D layered materials.

Among the 2D layered anisotropic materials, black phosphorus (BP) has a wide thickness-tunable direct bandgap (∼0.3 eV of monolayer to 2 eV of bulk) and high intrinsic mobility, with a puckered orthorhombic structure of a *Cmca* space group symmetry (see Fig. 3), which makes it a promising core material for next-generation (opto)electronic devices.^31,62–68^ In the atomic layer, each phosphorus atom in BP is connected to three adjacent phosphorus atoms, leading to two distinguishing defined directions: armchair and zigzag directions along the *x* and *y* axis, respectively. The highly anisotropic crystal lattice gives rise to its anisotropic in-plane electrical, optical, and phonon properties. Tellurene is another elementary in-plane anisotropic semiconductor, which is comprised of non-covalently bound parallel Te chains. Tellurene crystallizes in a structure composed of Te atomic chains in a triangular helix that are stacked together *via* van der Waals forces in a hexagonal array. In this structure, Te atoms form covalent bonds only to the two nearest neighboring Te atoms in the helical chain as shown in Fig. 3. The band gap of tellurene is also thickness-tunable varying from nearly direct 0.31 eV (bulk) to indirect 1.17 eV (2 L). Moreover, compared with BP, 2D tellurene also exhibits an extremely high hole mobility (∼10^5^ cm^2^ V^−1^ s^−1^) but has a better environmental stability. Tellurene, therefore, is expected to rival black phosphorus in many applications.^15,60,69–74^

### 2.2 Binary IV–VI chalcogenides

Similar to BP, the anisotropic layered IV–VI metal monochalcogenides (MX, M = Ge, Sn; X = S, Se, *etc.*) also possess puckered orthorhombic (distorted NaCl-type) crystal structure and exhibit high Grüneisen parameters, which give rise to ultralow thermal conductivities and exceptionally high thermoelectric figures of merit.^12,75^ In addition, their low-symmetry crystal structures can lead to highly anisotropic behaviors manifested in, such as, the in-plane anisotropic carrier's mobility,^76–78^ photoresponse,^42,79–82^ and Raman intensity.^78,83,84^ Conventionally, the zigzag accordion-like projection is defined as *x*-axis and *y*-axis denoting the armchair direction. Theoretical calculations have predicted the valley-dependent transport excited by linearly polarized light,^85^ reversible in-plane anisotropy switching by strain or electric field,^86^ and anisotropic spin-transport properties.^87^ Lin *et al.* demonstrated valley-dependent absorption excited by linearly polarized light.^88^ Beyond that, in the bulk forms, SnSe and SnS exhibit both multi-valley features at valence bands and very low thermal conductivities, which result in high thermoelectric anisotropic properties.^12,75^

Germanium disulfide (GeS_2_) and germanium diselenide (GeSe_2_) are other typical layered materials among binary IV–VI chalcogenides. Monoclinic β-GeSe_2_ is the most stable phase among all the GeSe_2_ phases with relatively lower lattice symmetry and exhibits in-plane anisotropic behaviors.^56,89,90^Fig. 3 shows the crystal structure of GeS_2_ and β-GeSe_2_. Unlike BP with strong interlayer coupling, the interlayer interactions of GeS_2_ and β-GeSe_2_ are relatively weak.^91^ Owing to the high stability under ambient environment and large direct bandgap, GeS_2_ and β-GeSe_2_ are promising candidates for short-wave photodetection.

### 2.3 Group IV–V compounds

Group IV–V compounds, silicon and germanium phosphides and arsenides, (*e.g.*, silicon phosphide (SiP), germanium phosphide (GeP), silicon arsenide (SiAs), germanium arsenide (GeAs), and germanium diarsenide (GeAs_2_)), are another family of low-symmetry layered materials.^92–95^ It is reported that they are crystallized into different layered structures with either orthorhombic (*Cmc*2_1_ space group, SiP,^44^ Pbma space group, SiP_2_ and GeAs_2_ (ref. 45)) or monoclinic (*C*2/*m* space group, GeP, GeAs and SiAs) symmetries.^52,96,97^ All these IV–V binary compounds are semiconductors with band gaps of 0.52–1.69 eV. In analogy to the transition metal dichalcogenides, the interactions between the layers are weak.^98^ People have also investigated the in-plane anisotropic optical, electrical, and optoelectrical properties of them due to the highly anisotropic dispersions of the band structures. Both theoretical calculations and experiments have revealed that 2D SiP has a widely tunable direct band gap (1.69–2.59 eV), high carrier mobility (2.034 × 10^3^ cm^2^ V^−1^ s^−1^) similar to BP and fast photoresponse.^44,99,100^ Moreover, GeAs and GeAs_2_ have been proved to be promising in thermoelectric materials by theoretical calculations and experiments.^44,99,100^

### 2.4 Transition metal dichalcogenides (TMDCs)

TMDCs have attracted increasing research interest due to their attractive physicochemical properties.^101–104^ Low level of in-plane crystal symmetry can also occur in TMDCs, such as 1T′-molybdenum disulfide (MoS_2_), Td-molybdenum ditelluride (MoTe_2_), Td-tungsten ditelluride (WTe_2_), rhenium disulfide (ReS_2_), and rhenium diselenide (ReSe_2_).^32,46,48,105,106^ Stable metallic 1T′-MoS_2_ (distorted octahedral MoS_2_) can be obtained and crystallizes in the orthorhombic crystal structure (*Pmn*2_1_). Unlike the trigonal prismatic (2H) or octahedral (1T) structure of MoS_2_, each Mo atom in 1T′-MoS_2_ is linked with six sulfur atoms and connects with two adjacent Mo atoms.^107^ Based on the distinct phased-induced anisotropy in 1T′-MoS_2_, people have investigated its anisotropic electrical transport properties and electrocatalytic performance.^46^ As for Td-MoTe_2_ and Td-WTe_2_, the Td phase shares the same in-plane crystal structure with the 1T′ phase but stacks vertically in a different way as depicted in Fig. 3. Td phase MoTe_2_ can be regarded as the distortion of MoTe_2_ along the *a*-axis. From Fig. 3, we can see that each Mo(W) atom bonds to two adjacent Te atoms, leading to the formation of Mo(W) chains along the *a*-axis, perpendicular to the in-plane *b*-axis and the interlayer *c*-axis. Besides the in-plane anisotropic properties, Td-MoTe_2_ and Td-WTe_2_ are also good candidates of type-II Weyl semimetals, which present a large amount of novel physical properties to be undiscovered.

Unlike MoS_2_ with hexagonal structures, group VI TMDCs with rhenium atoms (ReX_2_, X = S, Se) have distorted CdCl_2_ layer structure (denoted 1T′ phase, see Fig. 3) leading to triclinic symmetry and large in-plane anisotropy.^108,109^ In contrast to the 1T phase, the 1T′ phase displays covalent bonding between the nearest Re atoms. The covalent bonded Re atoms form diamond-like pattern leading to quasi one-dimensional Re chains.

### 2.5 Transition metal trichalcogenides (TMTC)

Group IV transition metal trichalcogenides MX_3_ are composed of transition metals M belonging to either group IVB (Ti, Zr, Hf) or group VB (Nb, Ta) and chalcogen atoms, X, from group VIA (S, Se, Te).^110,111^ The MX_3_ crystal structures (see Fig. 3) can be described as the stacking of individual chain units with the same orientation. Parallel neighbor chains are formed by sequential triangular prisms, where M and X atoms are respectively placed at the corners. These parallel chains in the same quasi-layer are bonded one to another with weak van der Waals interaction. Therefore, each layer of MX_3_ consists of unique quasi-1D chain-like structure and contributes to its anisotropic properties. In particular, titanium trisulfide (TiS_3_) that crystallizes in the monoclinic crystal structure (*P*2_1_/*m*) with two formula units per unit cell has a direct bandgap of 1.13 eV. Apart from the in-plane electrical anisotropy, TiS_3_ also exhibits ultrahigh efficiency of visible photoresponse, which makes it a suitable material for polarized photodetectors.^112^ In addition, NbS_3_ (triclinic structure), NbSe_3_ (monoclinic structure), and TaS_3_ (orthorhombic structure) also present the formation of charge density waves (CDW) and superconductivity at low temperature.^113,114^

### 2.6 Group III–VI compounds

Layered III–VI semiconductors, such as GaSe and InSe, are of wide interest due to their strong exciton peaks at room temperature absorption edge, large non-linear effect, and high intrinsic carrier mobility.^115–117^ They open up more possibilities for applications in non-linear optics and electronics. In contrast to GaSe, gallium telluride (GaTe) crystallizes in the monoclinic system with space group (*C*2/*m*) and one-third of the Ga–Ga bonds lies in the plane of the layer, as shown in Fig. 3. These bonds are perpendicular to the *b*-axis and lead to in-plane anisotropic physical properties.

Bulk TlSe crystallizes in a tetragonal structure with the space group of *I*4/*mcm*. Two thallium ions, monovalent Tl^+^ and trivalent Tl^3+^, exist in the crystal-line structure. The trivalent Tl^3+^ ions form chains of tetrahedral bonds disposed along the tetragonal axis, while the monovalent Tl^+^ ions are located between the chains and are held together by weak coupling interaction.^61^

### 2.7 Ternary transition metal chalcogenides

Nowadays, many 2D ternary transition metal chalcogenides (*i.e.*, Ta_2_NiS_5_, TaIrTe_4_) have been successfully fabricated and are good candidates for excitonic insulator and type II Weyl semimetals.^49,118,119^ In particular, the crystal structure of bulk Ta_2_NiS_5_ is shown in Fig. 3. It crystallizes in the orthorhombic structure with the space group *Cmcm*. The octahedral coordinated Ta chain and the tetrahedral coordinated Ni chain form one-dimensional structures along the *a*-axis and stack along the *c*-axis in the order of Ta–Ni–Ta. The NiS_4_ and TaS_6_ units are formed by coordination with the nearest-neighbor S atoms arranged along the *c* axis with NiS_4_ units, which are separated by two TaS_6_ units. Therefore, the different arrangement of chains in the layer gives rise to the one-dimensional characteristic.^120^

### 2.8 Group V_2_–VI_3_ compounds

V_2_–VI_3_ compounds, such as Bi_2_Te_3_ and Sb_2_Te_3_, have gained great interest and extensive research due to their striking thermoelectric properties and possibility to be topological insulators candidates.^121,122^ Some other V_2_–VI_3_ compounds such as Sb_2_S_3_, Sb_2_Se_3_, and Bi_2_S_3_ are composed of one dimensional covalently linked ribbons stacking along the *c*-axis by weak van der Waals interactions. Take Sb_2_Se_3_ for example; bulk Sb_2_Se_3_ was recently studied as a light sensitizer in photovoltaic devices due to its narrow direct band gap of about 1.1–1.3 eV, which crystallizes in an orthorhombic structure with the space group *Pnma*. It consists of staggered, parallel layers of 1D (Sb_4_Se_6_)_*n*_ ribbons that are composed of strong Sb–Se bonds along the 〈001〉 direction. For the 〈100〉 and 〈010〉 directions, the ribbons are stacked owing to their van der Waals interactions.^43,123^

### 2.9 Others

MoO_2_ crystallizes in the monoclinic phase with the space group of *P*2_1_/*c* and its crystal structure is distorted to the rutile-type.^57^ This is because O atoms are closely packed into octahedrons and Mo atoms occupy half the space of the octahedral void, which results in the edge-sharing MoO_6_ octahedrons connected with each other and forms the distorted rutile structure. Although MoO_2_ has a typical wide band gap, the Mo–Mo metallic bonds give rise to metallic transport properties.^124,125^

The binary alkaline metal phosphide family MP_15_ (M = Li, Na, K) crystallizes in the triclinic phase with the space group of *P*1̄.^59^ It is demonstrated that the anisotropic carrier mobility ratio of single-layer MP_15_ is extraordinarily large (∼10^1^ to 10^2^) between the *x*- and *y*-directions.^126^ MP_15_ is composed of parallel units with two antiparallel rows of P tubes in one [P15] unit. In one [P15]-cell, one P atom has two adjacent P atoms and the other 14 P atoms have three adjacent P atoms, which causes a pentagonal arrangement cross-sectionally. This tubular phosphorus structure makes KP15 highly anisotropic.

As seen from Fig. 3, the low-symmetry layered 2D materials in the same crystal structure and space group exhibit similar physical properties, which is highly desirable and important for the analysis of anisotropic properties in low-symmetry layered 2D materials.

## 3. Fabrication methods

Mono- and few-layer low symmetry 2D materials could be produced by using either “top-down” or “bottom-up” approaches. Top-down approaches include mechanical or ultrasound-assisted liquid phase exfoliation from the single crystal bulk. Bottom-up approaches, whereby the low symmetry materials are grown layer by layer, involve physical vapor deposition (PVD), chemical vapor deposition (CVD), molecular beam epitaxy (MBE), as well as solution synthesis.

### 3.1 Bottom down

#### 3.1.1 Mechanical exfoliation

**Fig. 4:**
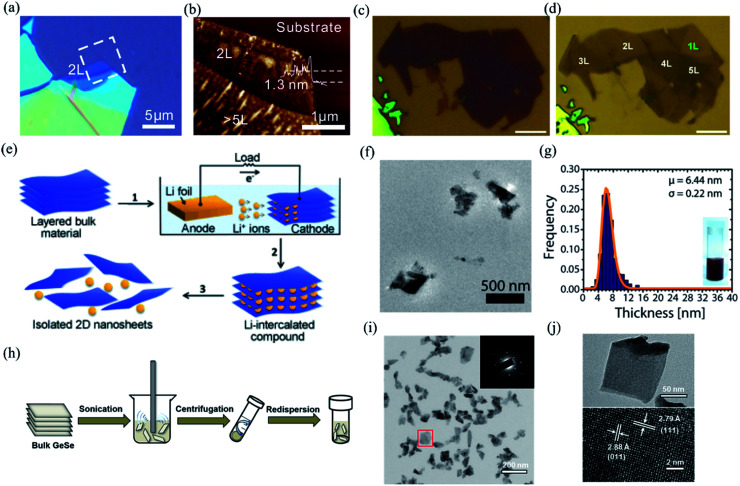
Optical image (a) and AFM image (b) of the mechanical exfoliated BP. Reproduced from ref. 18 with permission from American Chemical Society. (c and d) Optical images of multilayered BP before and after Ar^+^ plasma thinning. The scale bars are 5 μm. Reproduced from ref. 127 with permission from Tsinghua University Press. (e) Schematic of electrochemical lithiation process for the fabrication of layered 2D nanosheets from bulk material. Reproduced from ref. 128 with permission from Wiley-VCH. (f) Low magnification TEM image of the GeS nanosheets by LPE. (g) Thickness histograms of the as-exfoliated GeS nanosheets. Reproduced from ref. 129 with permission from American Chemical Society. (h) Schematic illustration of the ultrasonic assisted liquid phase exfoliation of GeSe. Large particles after sonication are removed by centrifugation at a moderate speed, while few-layer GeSe dispersed in the supernatant was precipitated at a higher speed and then redispersed in a different solvent. (i) TEM and SAED pattern (inset) of the GeSe nanosheet. (j) TEM and HRTEM image of the dispersed GeSe nanosheet. Reproduced from ref. 132 with permission from American Chemical Society.

Since Novoselov *et al.* successfully fabricated the first graphene flake using Scotch-tape in 2004,^1^ the mechanical exfoliation method has become commonly used to get few-layer single crystalline flakes of 2D materials due to the weak van der Waals interlayer interaction. In general, mechanical exfoliation is used to prepare monolayer or few-layer 2D materials by applying an adhesive tape to cleave bulk crystals repeatedly. Because of the as-cleaved clean surfaces and high crystallinity, the few-layer samples produced by mechanical exfoliation exhibit extraordinary physical properties. However, the exfoliated 2D materials still have some problems to be solved. Take BP for example; the pure exfoliated few-layer BP (see Fig. 4(a) and (b)) is relatively small sized with about 5 μm^2^ size.^18^ As shown in Fig. 4(c) and (d), although with the help of Ar^+^ plasma during the exfoliated process,^127^ the size of the monolayer BP can only reach 15 μm^2^, which is still far from our goals of large-scale fabrication, and well controlled morphology and edges of 2D materials.

#### 3.1.2 Liquid phase exfoliation (LPE)

One of the methods of LPE is chemically exfoliating the nanosheets of layered materials from the bulk powders with a solvent-free method by lithium intercalation. The powders are submerged in a lithium-containing solution such as *n*-butyllithium for days and lithium ions can intercalate in-between the layer space of the bulk material. Then, the nanosheets are separated when the intercalated ions are exposed to water. The schematic process is shown in Fig. 4(e).^128^ However, the disadvantage of ionic intercalation is that the nanosheets might be damaged during the process. The TEM image and thickness histograms of the GeS nanosheets produced by LPE are depicted in Fig. 4(f) and (g).^129^ Nowadays, ultrasonic assisted liquid phase exfoliation (UALPE)^130,131^ is being utilized to provide scalable production of 2D materials. The schematic illustration of UALPE is clearly shown in Fig. 4(h).^132^ In this method, the cavitation bubbles and shear force produced from the propagation of sonication waves could break the relatively weak van der Waals force between the layers without breaking the strong covalent intra-layer bindings. Therefore, this method can produce minimum defects on the as-exfoliated nanosheets due to the non-chemical and non-covalent interaction between the material and the liquid. The TEM and HRTEM images of the as-exfoliated GeSe are shown in Fig. 4(i) and (j), which show the high degree of crystallinity in the unbroken GeSe samples.^132^

### 3.2 Bottom up

#### 3.2.1 Physical vapor deposition (PVD) and chemical vapor deposition (CVD)

2D layered materials are foreseen to be the next-generation multifunctional materials, such as high-speed electronics and flexible optoelectronics, which compels researchers to fabricate 2D layered materials at the wafer scale. Because the bottom down approach can only produce the sheets at a micrometer scale, exploring the bottom up method, which has great potential to get a sizable 2D sample, is necessary. Up to now, many kinds of 2D layered materials have been fabricated through bottom up methods, including physical vapor deposition (PVD), chemical vapor deposition (CVD), molecular beam epitaxy (MBE), and atomic layer deposition (ALD).^133–136^

**Fig. 5:**
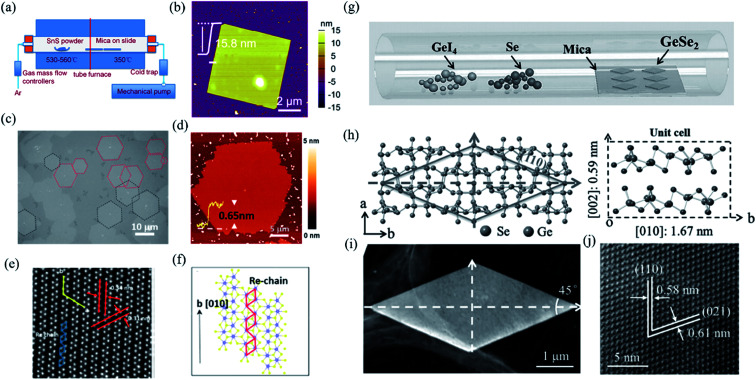
(a) Schematic illustration of the PVD growth system. (b) The AFM image of the as-grown SnS nanoplate transferred onto SiO_2_ substrates. Reproduced from ref. 137 with permission from American Chemical Society. (c) The optical image of the oriented hexagonal monolayer ReS_2_ in two distinct directions (red and black dashed lines) grown on the c-cut sapphire substrates by the CVD method. (d) The AFM image of the hexagonal monolayer ReS_2_. (e) HRSTEM images taken from the ReS_2_ monolayers showing the quasi-1D nature of the synthesized monolayers and (f) schematic of ReS_2_ monolayers along the *b*-axis lattice directions. Reproduced from ref. 138 with permission from American Chemical Society. (g) Schematic of the CVD method for the growth of GeSe_2_. (h) Crystal structure as determined by XRD. Left: looking down the *c*-axis, showing the (110) plane system. Right: unit cell of GeSe_2_, including some lattice parameters. (i) Low-magnification TEM image of the as-grown GeSe_2_ flake, and (j) the corresponding HRTEM image. Reproduced from ref. 90 with permission from Wiley-VCH.

In contrast to bottom down techniques, the bottom up PVD or CVD methods can not only fabricate the 2D layered materials at a large scale and with controllable thickness but also maintain the extraordinary quality, which is desirable for both fundamental research and device applications. For instance, Tian *et al.* have developed a PVD method, whose schematic instrument is shown in Fig. 5(a), to obtain rhombic SnS nanoplates with different thickness (6–20 nm).^137^ The AFM image of a 2D SnS nanoplate is shown in Fig. 5(b), which indicates good surface roughness and crystalline degree. Wu *et al.* recently improved the CVD synthesis of ReS_2_ monolayers onto [0001] (c-cut) sapphire substrates and produced highly crystallized ReS_2_ domains with well-defined structural anisotropy (see Fig. 5(c) and (d)).^138^ As shown in Fig. 5(e), the HRSTEM image of the monolayer ReS_2_ clearly shows the quasi-1D nature of the synthesized monolayer. The schematic depiction of the lattice directions during the growth of the monolayer is shown in Fig. 5(f). They found that the sapphire substrate can effectively control the shape, thickness, crystallinity, and structural anisotropy of the as-grown ReS_2_ flakes. Similarly, Zhou *et al.* have successfully produced single-crystalline rhombic β-GeSe_2_ flakes using van der Waals epitaxy and a halide precursor, which is shown in Fig. 5(g). The left of Fig. 5(h) shows the crystal structure of β-GeSe_2_ when looking down the *c*-axis. The angle between the (110) and (−110) planes is 45°. The unit cell of monoclinic crystalline GeSe_2_ along the *a*-axis is shown in the right of Fig. 5(h). It is interesting that the morphology of the flakes, as seen using low magnification (Fig. 5(i)), are consistent with the rhombic features of the molecular structure. Through high-resolution TEM (HRTEM) in Fig. 5(j), the high quality of the GeSe_2_ rhombic flakes were confirmed with the lattice fringes of the (110) and (021) planes that were measured to be 0.58 and 0.61 nm, respectively.^90^

#### 3.2.2 Solution synthesis

**Fig. 6:**
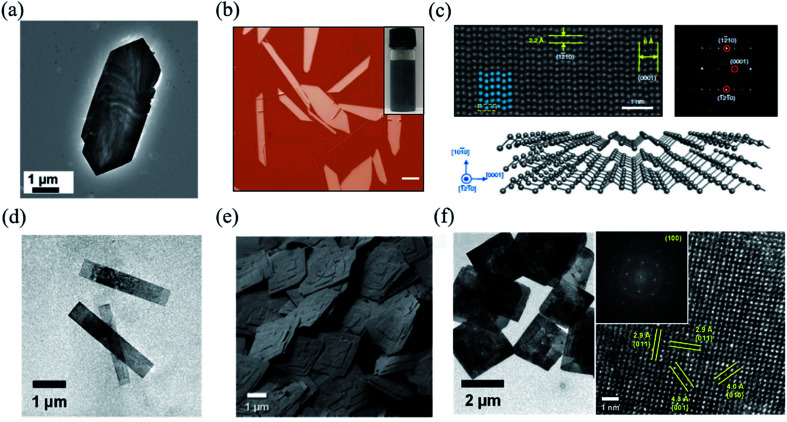
(a) TEM image of a single GeS nanosheet. Reproduced from ref. 142 with permission from Royal Society of Chemistry. (b) The optical image of solution-grown Te flakes. The inset is the optical image of the Te solution dispersion. The scale bar is 20 μm. (c) HAADF-STEM image of tellurene. False-coloured (in blue) atoms are superimposed on the original STEM image to highlight the helical structure. The upper right is the diffraction pattern of tellurene. The bottom is the illustration of the structure of tellurene. Reproduced from ref. 72 with permission from Nature Publishing Group. (d) TEM images of the colloidal SnS nanoribbons. (e) SEM images of the SnS square nanosheets dispersed on a substrate, indicating the high morphological uniformity of individual crystals within the colloidal solution. (f) The left shows the TEM images of μm-scale 2D colloidal SnS square nanosheets. The right is the HRTEM image of a single SnS nanoribbon and (inset) the resulting FFT, both of which reveal that they are single-crystalline with a surface that can be indexed to α-SnS (100). Reproduced from ref. 143 with permission from American Chemical Society.

As for PVD or CVD techniques, the crucial conditions for the nucleation and growth of 2D layered materials are at high temperature, controllable growth atmosphere, and appropriate epitaxial substrates, which limit the facile growth of 2D layered materials. A promising alternative to gas-phase deposition is solution-based synthetic strategies owing to its low demanded growth temperature and substrate-free growth process. Therefore, one can simply disperse the as-fabricated freestanding 2D layered materials and make straightforward flexible devices, assemblies, and thin films through means such as inkjet printing, spray coating, or roll-to-roll processing.^139–141^ In addition, the sizes and thicknesses of 2D layered materials can be effectively modulated by controlling the ratio of the precursors as well. Consequently, bottom-up solution-phase syntheses of 2D layered materials lend themselves promising commercial methods. For example, researchers have successfully synthesized and separated GeS, GeSe, tellurene, and colloidal SnS nanosheets from solution.^72,142,143^ Their corresponding characterizations are clearly shown in Fig. 6. The high degree of crystallinity and large-scale production demonstrate that solution-based synthetic strategy is one of the promising and desirable methods for manufacturing applications and devices in the future.

## 4. Characterization

The low-symmetry crystal structures and anisotropic band structures of highly asymmetric 2D layered materials enable their strong optical anisotropy. In order to rapidly and directly detect and characterize the optical anisotropy of the low-symmetry 2D layered materials without destroying the samples, the azimuth-dependent reflectance difference microscopy (ADRDM), angle-resolved polarization Raman spectroscopy (ARPRS), and polarization-dependent absorption spectroscopy (PDAS) are effective detection techniques.^96,112^

### 4.1 Polarization-dependent absorption spectroscopy (PDAS)

**Fig. 7:**
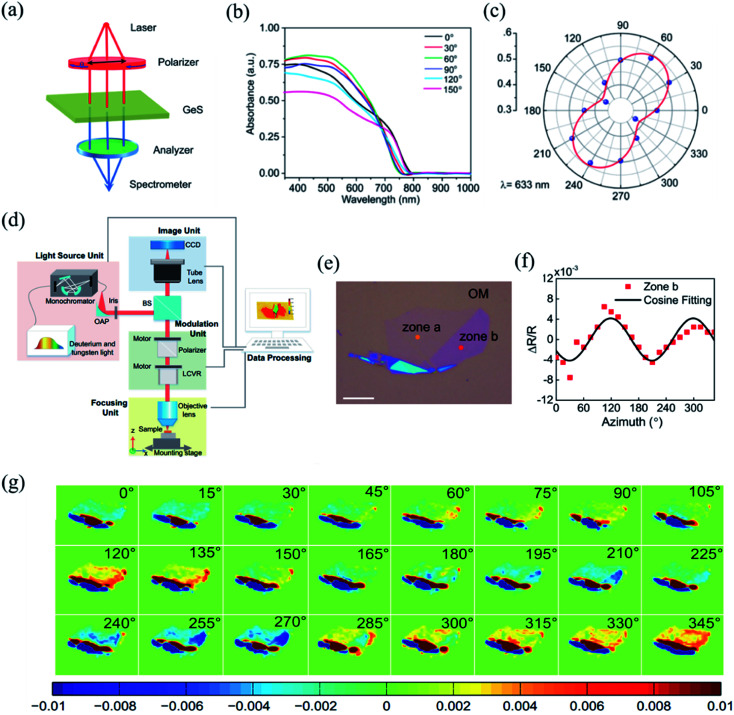
(a) Schematic of PDAS. (b) PDAS of the GeS flake with the spectral range 300–1000 nm. (c) Polar plot of the absorbance of the GeS flake at the wavelength of 633 nm. Reproduced from ref. 145 with permission from American Chemical Society. (d) Scheme of azimuth-dependent reflectance difference microscopy (ADRDM). (e) Optical image of a BP flake on the Si/SiO_2_ substrate. (f). Azimuth-dependent Δ*R*/*R* of zone b. (g) All the azimuth-dependent RDM images. Reproduced from ref. 148 with permission from Royal Society of Chemistry.

The detection principle of PDAS is to directly measure the difference of light absorption, which makes it a reliable method for the identification of crystalline orientation.^32,144–146^ The scheme of the PDAS measurements is displayed in Fig. 7(a). Firstly, the few-layer 2D materials are exfoliated and transferred on a quartz substrate. Then, the incident light beam is focused onto the flake and the inverted microscope is used to collect the transmitted light. Simultaneously, a spectrometer equipped with a CCD camera can analyze the intensity of transmitted light. If the anisotropic reflection can be neglected, the absorbance (*A*) is equal to ln(*I*_0_/*I*), where *I*_0_ and *I* are the light intensities transmitted through the quartz substrate nearby the flake location and through the flake, respectively. For example, Li *et al.* carried out the PRAS measurements of the multilayered GeS flake by rotating the direction of the probe light's polarization from 0° to 180°.^145^ The anisotropic absorption of GeS is clearly seen in Fig. 7(b) and the polar plot of absorption as a function of degree of polarization is shown in Fig. 7(c), thus presenting the linear dichroic characteristics of GeS. Since the polarization-dependent absorption spectroscopy only considers the electron–photon interaction, it is a reliable way to identify the crystalline orientations. Angle-resolved polarization Raman spectroscopy is another choice besides PDAS. However, it involves both electron–photon and electron–phonon interactions, which makes direct detection of crystalline orientation complicated.

### 4.2 Azimuth-dependent reflectance difference microscopy (ADRDM)

The detection principle of ADRDM is to directly measure the difference in the normalized reflectance (Δ*R*) between two arbitrary orthogonal directions in the surface plane (*a* and *b*) when the sample is illuminated by polarized light, which can be defined as:^147^1
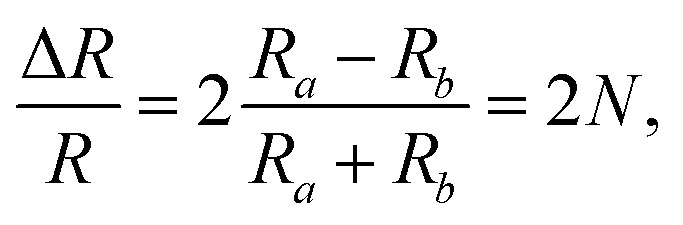
where *R*_*a*_ and *R*_*b*_ are the reflectance rate along *a*- and *b*-directions. The dimensionless value *N*(*θ*) alters as the incident direction of linearly polarized light changes, which can be described as:2
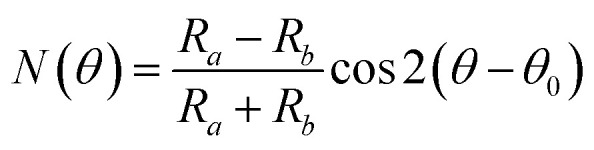
where *R*_*a*_ and *R*_*b*_ are the reflectance rate along the *a*- and *b*-directions of low-symmetry crystals, and *θ* and *θ*_0_ denote the azimuthal angles of the incident light and a direction of the sample, respectively. By plotting the *N*(*θ*) as a function of the azimuthal angle *θ*, the crystalline orientation of the low-symmetry crystals can be easily identified by according to the extreme values of the *N*(*θ*). From the equation, we can get that the maximum and minimum RD signals correspond to the high and low reflectance axes of the sample, respectively. In particular, ADRDM can collect *N*(*θ*) at all the pixels in the field and directly visualize the optical anisotropic contrast, which is especially useful for tiny sized 2D flake obtained from mechanical or liquid phase exfoliation. The scheme of ADRDM is shown in Fig. 7(d).^148^ Take BP for example; a typical optical image (OM) of exfoliated BP on the Si/SiO_2_ substrate is shown in Fig. 7(e). The Δ*R*/*R* values of BP in zone *b* as a function of the azimuthal angle *θ* of the incident light is displayed in Fig. 7(f), which shows a cosine function dependent. With the ADRDM result, the BP flake has two extreme Δ*R*/*R* directions of 115° and 205°, respectively. All the RDM images at different angles are depicted in a color scale in Fig. 7(g). However, even though the ADRDM measurement is a reliable technique for detecting the crystal orientations, the interference effect between the interfaces must be taken into account when the BP sheet is placed on a multilayer substrate (*e.g.*, SiO_2_/Si) because the interference effect will disturb the reflection signals and cause a reversed result.

### 4.3 Angle-resolved polarization Raman spectroscopy (ARPRS)

Based on group theory, from Raman tensors and density functional theory (DFT) calculations, the intensity of Raman signals can be quantitatively expressed as:^149^

**Fig. 8:**
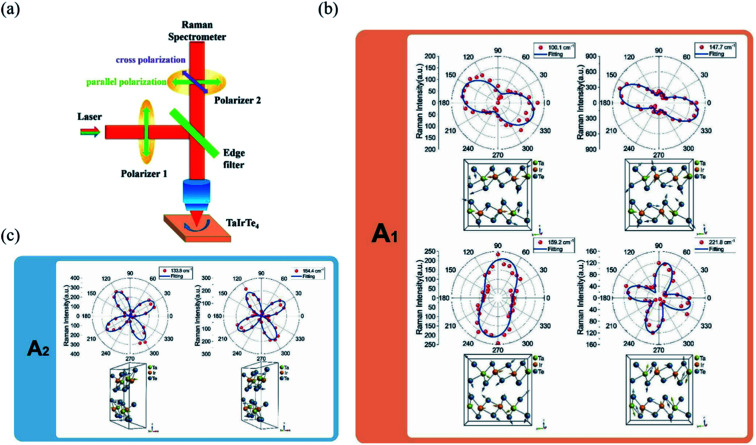
(a) Schematic of the angle-resolved polarized Raman spectroscopy of TaIrTe_4_ samples. Raman intensities of (b) A_1_ and (c) A_2_ modes as a function of the sample rotation angle and the corresponding phonon modes in the atomic view. The scattered dots are the experimental data and the solid lines are the fitting curves. Reproduced from ref. 49 with permission from Wiley-VCH.

3 *I* ∝ |*e*_i_***R****e*_s_|^2^

where *e*_i_ and *e*_s_ are the unit polarization vectors of the incident and scattered light, and ***R*** is the Raman tensor for a certain vibrational mode. For incident light, *e*_i_ = (cos *θ*, sin *θ*, 0), where *θ* is the angle between the incident light polarization and one crystalline orientation of the material. The schematic illustration of the angle-resolved polarized Raman spectroscopy is shown in Fig. 8(a). For the scattered light in the parallel-polarized configuration, *e*_s_ = (cos *θ*, sin *θ*, 0), while in the perpendicular-polarized configuration, *e*_s_ = (−sin *θ*, 0, cos *θ*). Take TaIrTe_4_ for example; the Raman tensors of A_1_, A_2_, B_1_, and B_2_ modes can be expressed as:^49^4
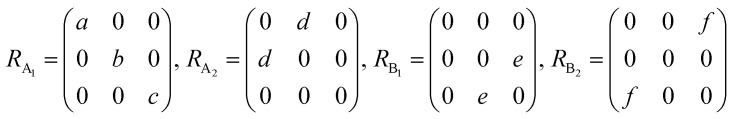
where *a*, *b*, *c*, *d*, *e*, and *f* are the tensor elements determined by the cross section of Raman scattering.^49^ Furthermore, the angle-dependent Raman scattering intensities of different modes can be expressed as:5
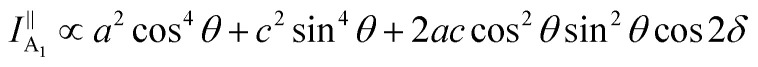
6
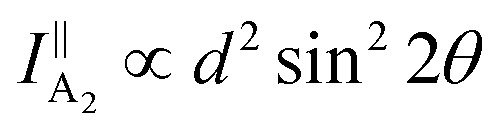
7
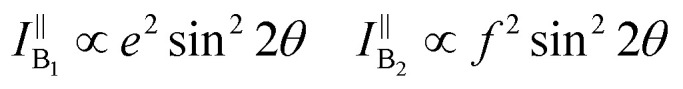


From the equations above and the measured Raman intensities of A_1_ and A_2_ modes shown in Fig. 7(c) and 8(b), we can see that the intensity of A_1_ mode varied in periods of 180° and 90° in parallel-polarized configuration, whereas the A_2_, B_1_, and B_2_ modes have 90° variation periods. Therefore, we can deduce the crystalline orientations of the low-symmetry materials by investigating the maximum and minimum intensities of A_1_ mode with a 180° variation period. The relative magnitude of matrix elements in A_1_, *a* > *c* or *a* < *c* determines whether the main axis is along the *a*-axis or *c*-axis. However, ARPRS alone cannot confirm the relative magnitude of *a* and *c*. In addition, because Raman scattering involves both electron–photon and electron–phonon interactions, the anisotropy of Raman scattering could be diverse at different detection conditions, such as the variable of laser wavelength and the thickness of sample.^96^ Therefore, combining ARPRS with other techniques such as high resolution TEM (HRTEM), PDAS, ADRDM, or angle-resolved DC conductance is an alternative method to confirm the crystalline orientations.

### 4.4 Angle-resolved DC conductance

**Fig. 9:**
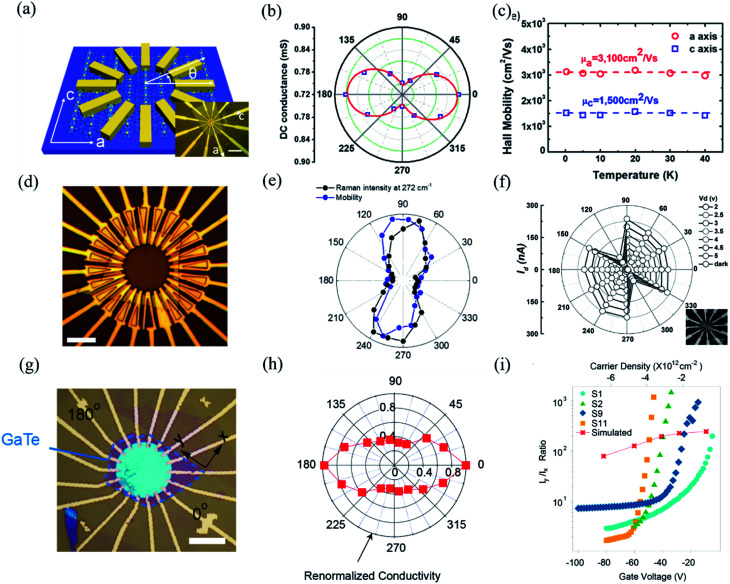
Angle-resolved DC conductance measurements. (a) Schematic illustration of the device structure of ZrTe_5_ flake. Inset: optical image of this device. Scale bar is 10 μm. (b) Angle-dependent DC conductance of the ZrTe_5_ flake. The data points are fitted with the equation: *σ*_θ_ = *σ*_x_ sin^2^ *θ* + *σ*_y_ cos^2^ *θ*. (c) Hall mobility along *a* and *c* axis at low temperatures. Reproduced from ref. 51 with permission from American Chemical Society. (d) Optical image of the transistors on the GeAs flake with electrodes spaced 15° apart for angle-resolved conductance measurement. (e) Polar plot of anisotropic field-effect mobility and Raman intensity at 272 cm^−1^ with orientation corresponding to optical image in figure (d). Reproduced from ref. 53 with permission from Wiley-VCH. (f) Angle-resolved current of the 10 nm thick Sb_2_Se_3_ nanosheet device at bias from 1 to 5 V. The inset is the optical image of the device. Reproduced from ref. 43 with permission from Wiley-VCH. (g) Optical image of a typical device made of 14 nm GaTe flake encapsulated in h-BN with electrodes spaced 20° apart. (h) Polar plot of normalized angle-dependent current at *V*_ds_ = 2 V and *V*_g_ = −80 V. (i) The electrical maximum anisotropic ratio *I*_y_/*I*_x_ extracted from different samples as a function of gate voltage. Reproduced from ref. 36 with permission from Nature Publishing Group.

Owing to the highly asymmetric crystal structure, the band dispersions along two perpendicular directions (*e.g.*, Γ–X and Γ–Y) and electron–phonon scattering may be strongly anisotropic. Therefore, the effective mass of the carriers along different crystalline orientations may differ a lot. According to the deformation potential theory, the anisotropy of effective mass gives rise to the anisotropy of the carrier's mobility *μ* and electrical conductivity *σ*. Consequently, by using the angle-resolved DC conductance measurement, one can independently determine the crystalline orientations for the low-symmetry layered materials. For instance, the electrical anisotropy of ZrTe_5_ was determined through the angle-resolved DC conductance measurement.^51^ In order to eliminate the geometric factors that might affect the current flow, the measured region should be circular. 12 electrodes were patterned uniformly and spaced at an angle of 30° along the directions, as shown in the inset of Fig. 9(a). Fig. 9(a) schematically illustrates the structure of the device. DC conductance measurements across each pair of diagonal electrodes at zero back gate bias were performed and the results are shown in Fig. 9(b). The angle dependent DC conductance fits well with the measured data using the equation:

8 *G*_θ_ = *G*_x_ sin^2^ *θ* + *G*_y_ cos^2^ *θ*

where *G*_x_ is the conductance along 〈100〉 direction and *G*_y_ is the conductance along the 〈001〉 direction. The DC conductance along the *a*-axis is 1.5 times larger than that along the *c*-axis. Therefore, we can identify the *a*- or *c*-axis by measuring the angle-resolved DC conductance. Moreover, the researchers also measured the carrier concentration and Hall mobility along two directions at low temperatures. The carrier concentrations remain constant along the two crystalline orientations, while the hole mobility along the *a*-axis is around 2 times larger than the *c*-axis, as shown in Fig. 9(c).

In the same way, Guo *et al.* also investigated the angle-resolved transport in multi-layered GaAs using the device shown in Fig. 9(d).^53^ An obvious anisotropic characteristics can be found by the angle dependent field-effect mobility, as shown in Fig. 9(d). The ratio of anisotropic mobility can reach as high as 4.8, which is comparable with black phosphorus and SnSe.^78,150^ Besides, from Fig. 9(e), we can see that the angle-resolved plot of Raman intensity at 272 cm^−1^ is very close to that of mobility, which means that the direction of maximum mobility (or conductance) is perpendicular to the *b*-direction of GeAs. As shown in Fig. 9(f), similar results can also be found in other low-symmetry layered materials such as Sb_2_Se_3_, whose ratio between maximum and minimum current is 16, which is the record of the in-plane anisotropic current (or conductance) ratio reported at room temperature.^43,54^

Recently, Wang *et al.* discovered that the angle dependent conductance can be effectively modulated by gate bias in few-layered semiconducting GaTe.^36^ The optical image of the device is shown in Fig. 9(g). By measuring the anisotropic DC conductance at *V*_g_ = −80 V, as shown in Fig. 9(h), one can identify that the maximum *I*_ds_ flow is in 0°, which is parallel to the *y*-axis, as marked in Fig. 9(g). It is striking that the ratio of anisotropic conductance (*I*_max_/*I*_min_) is gate-tunable and can reach as high as 10^3^ at *V*_g_ = −30 V, as shown in Fig. 9(i). The gate-tunable anisotropic conductance is probably due to the different ratio of transmission channels in *x*- and *y*-directions at diverse gate bias. By calculating the transmission coefficient, the researchers found that at low gate voltage (−9.1 V), there is almost no *x*-direction transmission channel in the scattering region between the source energy level and drain energy, while a sizable *y*-direction transmission is observed, resulting in a large anisotropic ratio at low gate voltages. In contrast, at high gate voltage (−82 V), the transmission is comparable in both *x*- and *y*-directions, thus greatly suppressing the anisotropic ratio in GaTe.

Beyond the results described above, researchers have also investigated the anisotropic carrier transport properties of other low-symmetry 2D materials by the angle-resolved DC conductance method as well. The predicted and measured anisotropic effective mass, mobility, and conductance of low-symmetry 2D materials are summarized and depicted in Table 1. We can see that the studies of anisotropic carrier transport properties of certain low-symmetry 2D materials are still missing. There is no doubt that one can fabricate anisotropic devices with higher performance if low-symmetry 2D materials with large anisotropy ratio of carrier transport were studied more deeply.

Based on the detection principles of different measurements mentioned above, we can see that PDAS and ADRDM are reliable ways to quickly and directly identify crystalline orientations without damaging the materials. However, if the 2D materials are extremely thin, the signals of PDAS are too weak to detect and resolve. In addition, the interference effect may cause a reversed result of ADRDM when the 2D materials are placed on a multilayer substrate (*e.g.*, SiO_2_/Si). ARPRS is another choice besides PDAS and ADRDM. However, it involves both electron–photon and electron–phonon interactions, which make the direct detection of crystalline orientation complicated and difficult. Meanwhile, the anisotropy of Raman scattering is strongly dependent on the laser wavelength and the thickness of the sample. Therefore, ARPRS might be restricted to analysis when it is compared with other techniques. In the end, even though angle-resolved DC conductance measurement can effectively identify the crystal directions, the procedure of fabricating the electrodes is complicated and time consuming.

## 5. Multifunctionality

### 5.1 Anisotropic magneto-transport properties

#### 5.1.1 Anisotropic magneto-resistance (MR)

Investigating the magneto-transport properties of materials could provide a more comprehensive understanding of their physical properties and insights into potential applications.^151^ Here, we review some recent reports on the anisotropic magneto-transport properties of low-symmetry layered 2D materials in order to explore the rich physics in them.

**Fig. 10:**
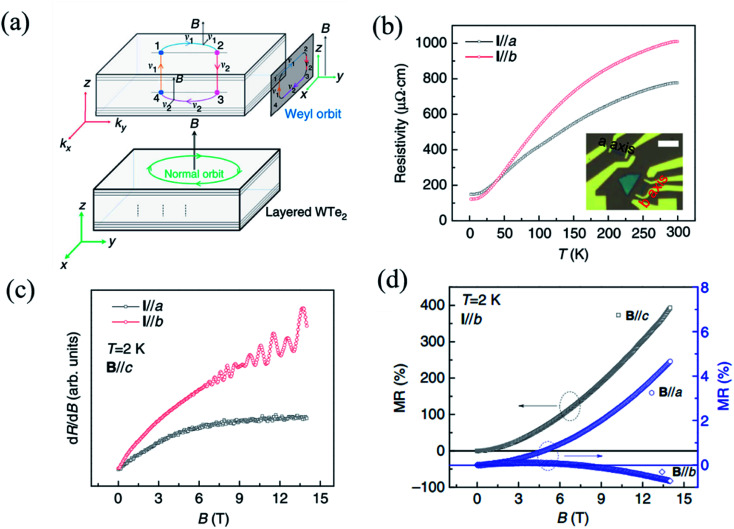
(a) Schematic of a Fermi-arc-induced Weyl orbit in a thin WTe_2_ nanoribbon, in which the magnetic field is along the *z*-axis (or *c*-) axis. The trajectory of the Weyl orbit in real space is in the *xz*-plane, and the Weyl orbit is plotted in a combination of real space and momentum space. (b) Temperature dependent resistivity of WTe_2_ nanoribbon with the current along the *a*- and *b*-axis. The inset is the optical image of these two ribbons and the scale bar is 5 μm. (c) Quantum oscillation in both the ribbons with the magnetic field parallel to the *a*- and *b*-axis. (d) Anisotropic MR data obtained along *a*, *b*, and *c* field directions at 2 K. Reproduced from ref. 154 with permission from Nature Publishing Group.

As the first predicted candidate for a type-II Weyl semimetal, Td-WTe_2_ has become an attractive topic owing to its exotic physical properties, such as huge non-saturated magnetoresistance (MR), chiral anomaly, and ultrahigh carrier mobilities.^13,152–154^ The non-saturable large MR and chiral anomaly of WTe_2_ are strongly related to its Td crystal structure. Recently, Li *et al.* have proved that WTe_2_ was indeed a type-II Weyl semimetal with topological Fermi arcs by observing the anisotropic chiral anomaly through magneto-transport measurements in one WTe_2_ nanoribbon.^154^ When the electric field is applied along the *k*_y_(*b*-)-direction and the magnetic field is applied along the *z*-(or *c*-) direction in the *b*-axis ribbon of WTe_2_, a closed Weyl orbit is formed (Fig. 10(a)) and corresponds to a trajectory in the *xz*-plane in real space. The temperature-dependent resistivity curves of the *a*-axis and *b*-axis ribbons shown in Fig. 10(b), which demonstrate the anisotropic transport properties in WTe_2_. A higher residual resistivity along the *a*-axis than that along the *b*-axis indicates that the average carrier mobility is smaller along the *a*-axis than along the *b*-axis (*σ* = (*n* + *p*)*eμ*), which is consistent with the transport anisotropy observed previously in bulk WTe_2_.^13,155^ In order to confirm the existence of a Weyl orbit (Fermi arcs), the authors measured the MR of both the *a*-axis and *b*-axis ribbon at 2 K, as shown in Fig. 10(c), where quantum oscillations can be observed in the *b*-axis ribbon, while those cannot be seen in the *a*-axis ribbon. Therefore, it is demonstrated that the quantum oscillations came from the Weyl orbit (Fermi arcs) instead of the trivial surface states. The disappearance of quantum oscillations in the *a*-axis ribbon can be attributed to the strong mobility anisotropy *μ*_a_ < *μ*_b_, which is supported by the data in Fig. 10(b). In addition, a negative MR induced by this chiral anomaly should be observed when a magnetic field is applied parallel to the tilted direction of the Weyl cones. Inversely, the positive MR emerges when the unsaturated electric field and the magnetic field are mutually vertical, and the current is parallel to the W-chain.^156,157^ The anisotropic MR curves measured at 2 K with *B*∥*a*, *B*∥*b*, and *B*∥*c* are shown in Fig. 10(d). Although the magnetic fields are perpendicular to the current in both cases, *B*∥*a* and *B*∥*c*, the positive MR ratio when *B*∥*a* is almost two orders of magnitude smaller than that when *B*∥*c* is consistent with a previous observation in bulk WTe_2_.^33^

Meanwhile, negative MR can be observed when *B*∥*b*. When magnetic field tilts slightly from the *E*-field direction, the absolute value of negative MR in the *b*-axis ribbon decreases quickly, while no negative MR can be observed in the *a*-axis ribbon. All these experimental data demonstrate that the Weyl points and Fermi arcs are found along the *y*-direction (*b*-axis) and are indeed induced by the chiral anomaly. This strongly anisotropic MR behavior is mainly ascribed to the strong anisotropy in the carrier mobility.^13,155^ Therefore, the measured anisotropic magneto-transport properties can indeed give evidence to the band structure of some low-symmetry 2D materials.

#### 5.1.2 Anisotropic nonlinear magneto-resistance (NLMR)

**Fig. 11:**
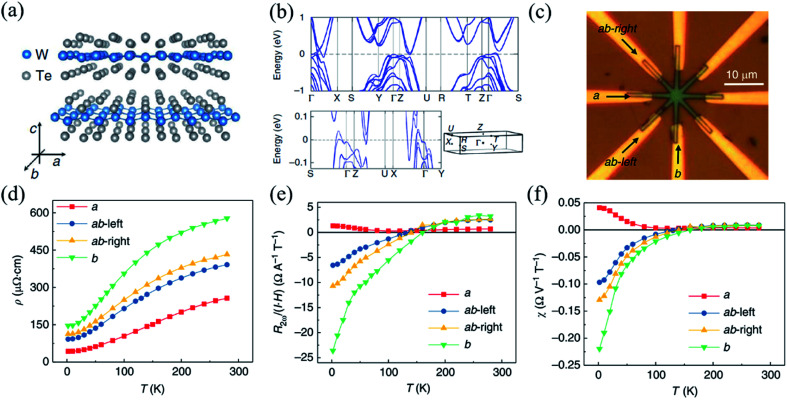
(a) Crystal structure of the layered WTe_2_ and its crystalline directions. (b) The calculated band structure of bulk WTe_2_, where the high symmetry *k* points are indicated in the 3D Brillouin zone sketched underneath. (c) Optical image of the device with arrows indicating the *a*- and *b*-directions. (d) Temperature dependent linear resistivity *ρ* along different crystalline directions. Temperature dependence of NLMR R_2ω_ normalized under unit current and magnetic field (e) and normalized under unit electric voltage and magnetic field (f) along different crystalline orientations. Reproduced from ref. 158 with permission from Nature Publishing Group.

Apart from the linear magneto-transport in Td-WTe_2_, recently, He *et al.* have also investigated the spin-dependent non-linear magneto-transport in Td-WTe_2_ to explore its spin-polarized bands and their interplay with Fermi surface.^158^ The crystal and calculated band structure of distorted Td-WTe_2_ are shown in Fig. 11(a) and (b). It is known that linear resistance is current-independent, while non-linear resistance is current-dependent. In the non-linear magneto-transport measurements, a low-frequency ac current was applied in the device and the second-harmonic longitudinal voltage was measured by lock-in techniques. Since there are strong local distortion of W ions in the Td-phase, as shown in Fig. 11(a), the non-linear magnetoresistance (NLMR) may be strongly anisotropic. Therefore, as displayed in the Fig. 11(c), four pairs of electrodes were patterned and spaced at an angle of 45 degree along the directions of *a*-, *b*-, *ab*-left-, and *ab*-right-axis, which were initially identified by polarized Raman spectrum. The values of linear resistivity along the *b*-axis are about three times larger than those along the *a*-axis at different temperatures, as measured and shown in Fig. 11(d). But for normalized NLMR under unit current (voltage) and magnetic field, the sign inverses with temperature when the current is along the *b*-axis, while it does not inverse with temperature when the current is along the *a*-axis, which is shown in Fig. 11(e) and (f). Such large anisotropic NLMR has not been reported in other materials before.^159–161^ By using the DFT calculations and tight-binding model, the authors found that the sign of the non-linear current was decided by the Fermi surface convexity and the strongly anisotropic NLMR was attributed to the low symmetry of the Fermi surface.

#### 5.1.3 Anisotropic weak localization (WL) effect

**Fig. 12:**
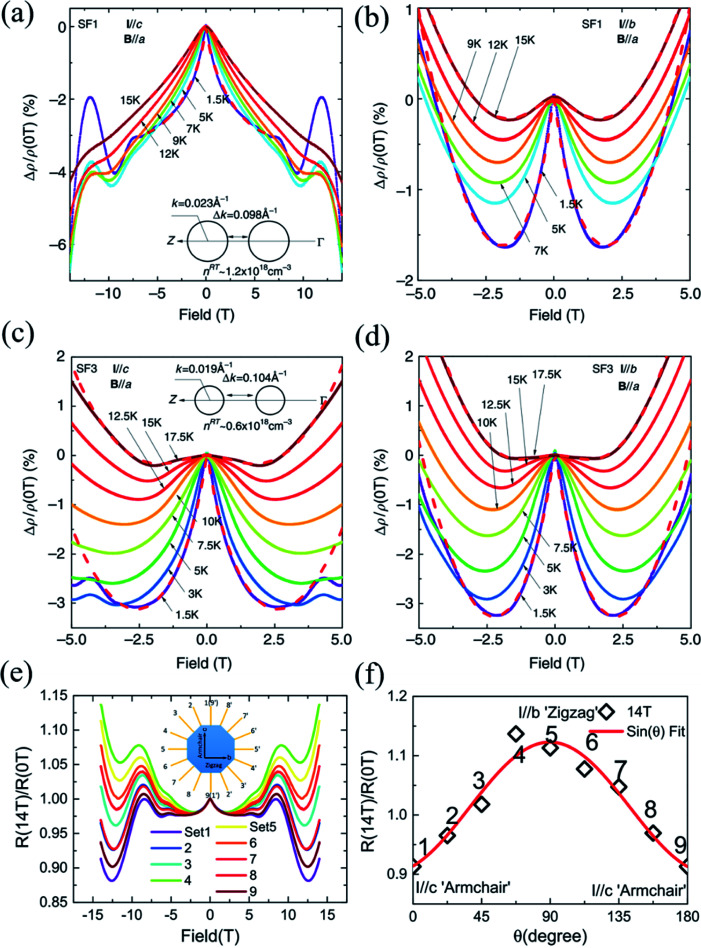
Negative MR in the SF1 sample for (a) *I*∥*c* and (b) *I*∥*b* at different temperatures, respectively. The low field MR in the SF3 sample at different temperatures when (c) *I*∥*c* and (d) *I*∥*b*. The low-field negative MR in the SF1 sample is more anisotropic than the SF3 sample. The insets of (a) and (c) show the sketches of the cross-sections of Fermi surfaces for the SF1 and SF3 samples, respectively. (e) Anisotropic MR for *I* applied along various directions and *B*∥*a*. (f) The normalized MR curves by the zero-field resistivity for better comparison. Note the exotic non-saturating behavior up to 14 T for *I*∥*c*-axis. Reproduced from ref. 162 with permission from Nature Publishing Group.

Through investigation of anisotropic magneto-transport properties, one can not only confirm the anisotropic chiral anomaly in Td-WTe_2_ but also investigate the anisotropic electronic structure in SnSe. Wang *et al.* have recently studied the highly anisotropic electronic structure of SnSe by combining angle-resolved photoemission spectroscopy with angular dependent magneto-transport measurements.^162^ The authors have synthesized several batches of SnSe single crystals using different growth methods, including self-flux (SF) and Bridgeman (BR). On account of the different conditions during growth, the amount of Se vacancy in SnSe crystals is varied and gives rise to different doping and concentrations of carriers. By analyzing the measured SdH oscillations and the Hall effect, the obtained concentration of SF1 sample is about two times larger than that of the SF3 sample. Both samples show metallic transport from *ρ*–*T* curves and weak localization (WL) at low temperatures (below 50 K). The charge transport in the samples are dominated by the multivalley Fermi surfaces of the pudding-mould shaped VB1, which can result in exotic quantum phenomena in p-SnSe. Therefore, the anisotropic MR of both SF1 and SF3 samples are measured for comparison. As shown in Fig. 12(a), when the magnetic field is perpendicular to the *b-c*-plane (*B*∥*a*-axis) and the current is along the armchair direction (*I*∥*c*-axis), the WL effect induced negative MRs is pronounced and does not show any sign of saturation up to 14 T.

However, such exotic behaviors of the *I*∥*c*-axis are in striking contrast to the MR behaviors when the current is parallel to the zigzag direction (*I*∥*b*-axis). The WL effect induced negative MRs are only dominant at low fields below 2 T before prevalence of positive MR, as shown in Fig. 12(b). However, the MR characteristics in the SF3 sample is less anisotropic than the SF1 sample, while the WL effect is more robust and dominant in the SF3 sample. From Fig. 12(c) and (d), we can see that the SF3 sample shows significantly larger low-field negative MR, which can reach as large as ∼ −3% at 2 T and 1.5 K, than the SF1 sample in the same configuration. But the magnitude of low-field negative MR does not differ much in comparison with the SF1 sample. As shown in the insets of Fig. 12(a) and (c), because the hole doping in the SF1 sample is about two times higher than that of the SF3 sample, the Fermi energy level is shifted downwards by about 5 meV, which reduces the separations between the two pudding-mould valleys. Thus, the momentum mismatch Δ*p* is compensated by the dipole field acceleration of hole carriers and the intervalley scattering is expected to be stronger in SF1 sample when *I*∥*c*. Generally, for non-relativistic fermions, the enhanced intervalley scattering gives rise to the suppression of WL because of the interruption of backscattering loops. Therefore, the WL effect induced negative MR is weakened in the SF1 sample and dependent on doping. Also, the in-plane anisotropic WL phenomena may be attributed to strong intervalley scattering along the ferroelectric dipole field direction (*c*-axis). Moreover, the anisotropic and non-saturating MRs can also be observed in the BR1 sample, as shown in Fig. 12(e) and (f).

#### 5.1.4 Anisotropic superconducting

**Fig. 13:**
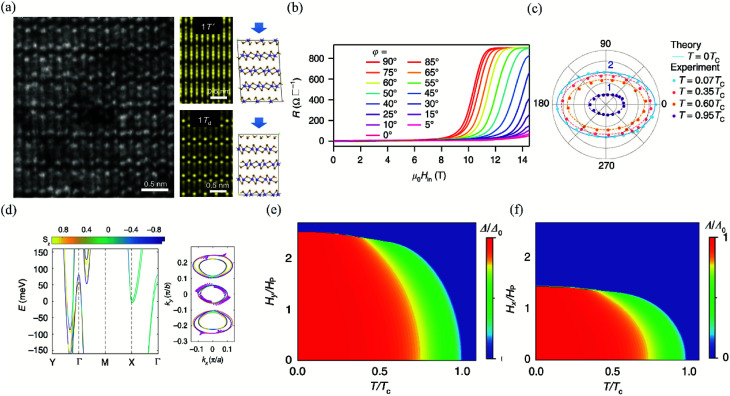
(a) The left is the STEM image of few-layer Td-MoTe_2_. The right is the simulated STEM images of few-layer MoTe_2_ in 1T′ and Td stacking viewed along the (001) axis, respectively. (b) MR of the 3 nm-thick MoTe_2_ device at *T* = 0.3 K (*T* = 0.07*T*_c_) with different in-plane tilted angles *φ*. (c) Angular dependence of the in-plane upper critical field normalized by Pauli limit *H*_c2_/*H*_p_. The experimental data are measured at 0.07*T*_c_, 0.35*T*_c_, 0.6*T*_c_, and 0.95*T*_c_, respectively. The theoretical value of *H*^∥^_c2_ at *T* = 0 K is plotted to show the two-fold symmetry consistent with the experimental data at low temperature. (d) The left is the first-principle calculations for the band structure of the bilayer Td-MoTe_2_. The right is the in-plane spin texture at the Fermi level. The temperature phase diagram for the superconducting state with anisotropic SOC under (e) *φ* = 90° and (f) *φ* = 0° oriented in-plane magnetic field, respectively. Reproduced from ref. 163 with permission from Nature Publishing Group.

Furthermore, Cui *et al.* have studied the anisotropic spin-orbital coupling (SOC) and demonstrated that the in-plane upper critical field in the superconducting few-layer Td-MoTe_2_ exceeded the Pauli limit in the whole in-plane directions.^163^ Through the atom-resolved STEM image of few-layer MoTe_2_ at a large scale, as displayed in Fig. 13(a), the Td phase of the CVD-grown MoTe_2_ could be unambiguously confirmed. The MR of the 3 nm-thick MoTe_2_ at 0.3 K (*T* = 0.07*T*_c_) with various in-plane tilted angle *φ* is shown in Fig. 13(b)*φ* is defined as the degree between *x*-axis of MoTe_2_ and the magnetic field. As we can see from Fig. 13(b), the superconducting transition moves from the lower magnetic field to the higher magnetic field when *φ* rotates from 90 degree to 0 degree, which clearly shows the anisotropy of superconducting. Besides, the in-plane upper critical field (*H*^∥^_c2_) in this few-layer MoTe_2_ also has an angular dependence at different temperatures, as shown in Fig. 13(c). The in-plane inversion asymmetry can induce strong SOC splitting and lead to effective Zeeman magnetic field with opposite out-of-plane direction at *K* and −*K* valleys of the Brillouin zone. Consequently, the spins of electrons in Cooper pairs are orientated by the effective Zeeman magnetic field and become insensitive to the external in-plane magnetic field.^164,165^ Therefore, the in-plane upper critical field of few-layer MoTe_2_ can exceed the Pauli limit *H*_p_ in the in-plane directions. In order to further confirm this interpretation, the band structure of the bilayer Td-MoTe_2_ as well as the anisotropic spin texture calculated by the first principle are presented in Fig. 13(d) and (e). The in-plane spin–orbit coupling (SOC) is highly anisotropic at the Γ pockets and the out-of-plane spin polarization dominates for the other two pockets. Moreover, the temperature phase diagrams for the superconducting state under different directions of the in-plane magnetic field are plotted by the mean field calculations for the pairing order parameter dependence on the in-plane magnetic field along *φ* = 90° and *φ* = 0° directions, as shown in Fig. 13(e) and (f). The obvious difference of *H*_c2_ along *φ* = 90° and *φ* = 0° directions signifies the in-plane anisotropic SOC at temperatures below *T*_C_ and agrees well with the trend of the experimental data in Fig. 13(b).

From the reported results above, the anisotropic magneto-transport measurements have been proven to be powerful and useful in the study of band structures and new physical phenomena of low-symmetry layered 2D materials.

### 5.2 Anisotropic optoelectronic properties

**Fig. 14:**
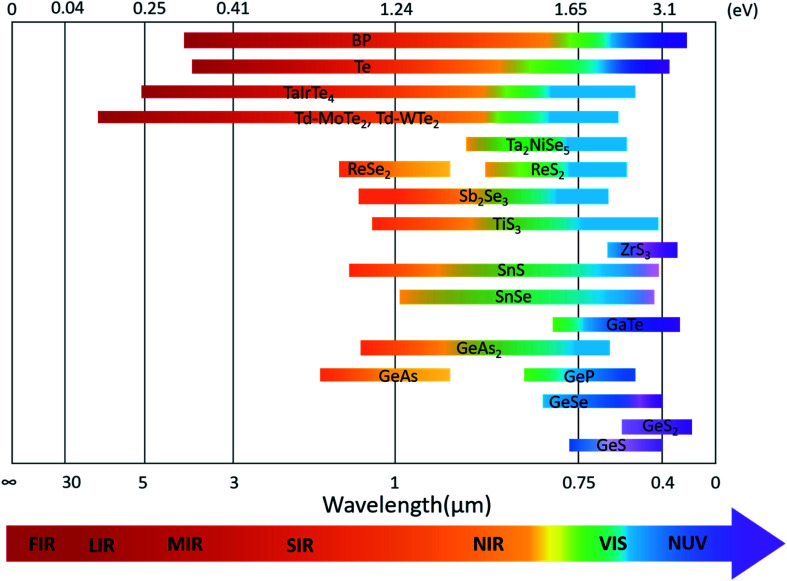
Band-gap values of various low-symmetry 2D materials and their corresponding absorption or detection range.

The optoelectronic properties of 2D layered materials are strongly related to the band gap and light absorption coefficient, which are depicted in Table 1. Similar to the conventional semiconductors, low-symmetry 2D materials, *e.g.*, black phosphorus, Td-MoTe_2_, tellurene, and ternary TaIrTe_4_, can also realize a wide response range across the electromagnetic spectrum because of their small bandgaps. The bandgap values of low-symmetry 2D materials and their corresponding detection range are summarized in Fig. 14.

Likewise, anisotropic optoelectronic properties can be introduced by reducing the lattice symmetry of layered materials. To explore the optoelectronic applications deeply, the detection of polarized light is exceptionally useful in several fields, including optical communication, remote sensing, and optical data storage.^166,167^ Since highly asymmetric arrangement of atoms can lead to anisotropic band dispersions, further leading to the anisotropic electronic and optical properties, and thus optoelectronic properties, the materials with anisotropic optoelectronic properties are promising candidates for polarization-sensitive photodetectors.^31,106,168,169^

**Fig. 15:**
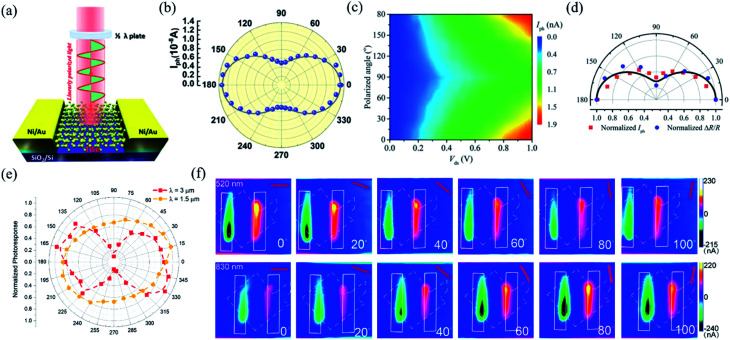
(a) Schematic of the polarization-sensitive photodetector. (b) Polar plots of the photocurrent of the TlSe flake as a function of *φ* at *V*_ds_ = 1 V (from 0° to 360° with a step size of 10°). Reproduced from ref. 61 with permission from American Chemical Society. (c) The polarization-dependent photocurrent of GeAs_2_ at different *V*_ds_. The light polarized along the *a*-axis is defined as 0° reference. (d) Polar plot of the normalized polarization-dependent photocurrent (red square) and reflectance contrast (blue circle) in GeAs_2_. Reproduced from ref. 45 with permission from Wiley-VCH. (e) Polar plots of the normalized polarization-dependent photocurrent of the 2D Te flake at wavelengths of 3 and 1.5 μm. Reproduced from ref. 73 with permission from American Chemical Society. (f) Polarization-dependent photocurrent mapping of the GeAs device at *V*_ds_ = 30 mV under the illumination of 520 nm and 830 nm laser, respectively, showing linear dichroic photodetection. Reproduced from ref. 170 with permission from American Chemical Society.

In order to investigate the anisotropic optoelectronic properties of low-symmetry materials, two-terminal phototransistors were fabricated, as schematically shown in Fig. 15(a).^61^ In the measurement, the polarized incident light was modulated by the *λ*/2 plate and changed at a step of one certain degree. Then, the photocurrents at different polarized degrees can be obtained and plotted. Fig. 15(b) shows the typical polarization-sensitive photoresponse of the 2D TlSe flake with two-fold symmetry axes.^61^ To characterize the magnitude of the linear dichromic photoresponse, a dichroic ratio *γ* = *I*_max_/*I*_min_ can be introduced. The larger the value of the dichroic ratio that is measured, the more sensitivity to the polarized incident light the material exhibits. Furthermore, it is of vital importance to figure out the origin of the polarization-sensitive photoresponse. Zhai *et al.* have recently measured the polarization-dependent photocurrent of the few-layered GeAs_2_ at different source-drain bias, as shown in Fig. 15(c).^45^ The authors also measured the polarization-dependent reflectance contrast of the sample and compared it with the trend of polarization-dependent photocurrent, as shown in Fig. 15(d). Both the photocurrent and reflectance contrast display similar polarization-dependent behavior, which manifests that the origin of polarization-dependent photocurrent is the sample's intrinsic linear dichroism. Javey *et al.* have investigated the polarization-dependent photoresponse of 2D Te nanoflakes.^73^ Surprisingly, the behavior of photoresponse as a function of polarization under the illumination of 1.5 and 3 μm laser is different, as shown in Fig. 15(e). Since Te has a direct band gap at 0.71 eV due to a strong absorption when the polarized light is along the direction of Te-chain and an indirect band gap at 0.31 eV owing to a weak absorption when the polarized light is perpendicular to the direction of Te-chain, the photoresponse of 3 μm (indirect band gap) is more anisotropic than that of 1.5 μm (direct band gap). Scanning photocurrent microscopy (SPCM) has been widely utilized for understanding the mechanism for the generation of photocurrent. In order to exclude the anisotropic collection of the photo-induced carriers, Yuan *et al.* have fabricated a ring-shaped electrode on the BP flake. By measuring the mapping of polarization-dependent photocurrent, they demonstrated that the mechanism of the photocurrent in BP is photothermoelectric effect and the dichroic ratio of BP was about 3.5 at 1200 nm.^31^ Typical mappings of polarization-dependent photocurrent in a GeAs flake by SPCM measurement are displayed in Fig. 15(f).^170^ As shown in Fig. 15(f), the photocurrent is predominantly generated near the contact between the electrodes and the sample, and has opposite sign at the two electrodes, from which we can deduce that the Schottky barriers are formed at the interface between the electrodes and the sample, and the mechanism of photocurrent is photovoltaic and photothermoelectric effect. Moreover, from Fig. 15(f), it is clearly seen that the maximum photoresponse direction under 520 nm light is along about 0°, while it differs by about 80° under 830 nm light. This interesting phenomenon may be related to the strongest absorption polarization reversing from *b*-axis to *a*-axis at 623 nm. Other low-symmetry layered 2D materials exhibit polarization-sensitive photoresponse as well. We have summarized the reported dichroic ratio of the low-symmetry layered 2D materials in Table 2.

**Table 2:** Some recently reported 2D-based anisotropic photodetectors and their performance parameters (N/A: not applicable)

Device	Responsitivity (*R*)	Detectivity (D*)	Response time	Dichroic ratio	Ref.
BP	518 mA W^−1^ at 3.4 μm	N/A	N/A	≈4 at 5 μm	223
	23A W^−1^ at 3.68 μm	N/A	N/A	N/A	224
	2A W^−1^ at 4 μm				
	≈11 A W^−1^ at 3.7 μm	6 × 10^10^ cm Hz^1/2^ W^−1^ at 3.8 μm	12.4 μs (980 nm)	>100 at (3.5 μm)	217
As_0.83_P_0.17_	216.1 mA W^−1^ at 2.36 μm	9.2 × 10^9^ jones at 2.4 μm	0.54 ms at 4.034 μm	3.88 at (1550 nm)	225
As_0.83_P_0.17_	190 mA W^−1^ at 3.4 μm	N/A	N/A	≈6 (3.4 μm)	226
As_0.91_P_0.09_	N/A	2.4 × 10^10^ cm Hz^1/2^ W^−1^ at 4.2 μm	N/A	N/A	217
Te	5.3 A W^−1^ at 1.5 μm	N/A	N/A	1.43 (1.5 μm)	73
	3 A W^−1^ at 3.0 μm			6.0 (3.0 μm)	
GeS	206 A W^−1^ at 633 nm	2.35 × 10^13^ jones at 633 nm	7 ± 2 ms	N/A	227
	6.8 × 10^3^ A at 500 nm	5.6 × 10^14^ jones at 633 nm	200 ms	N/A	81
	173 A W^−1^ at 405 nm	1.74 × 10^13^ jones at 633 nm	0.11 s	N/A	142
GeS_2_	N/A	N/A	N/A	2.1 (325 nm)	228
GeSe	4.25 A W^−1^ at 532 nm	N/A	N/A	1.09 (532 nm), 1.44 (638 nm), 2.16 (808 nm)	218
	870 A W^−1^ at 405 nm	1.12 × 10^13^ jones at 633 nm	0.15 s	N/A	142
	7.05 A W^−1^ at 633 nm	3.04 × 10^8^ jones at 633 nm	1 s	N/A	79
	1.6 × 10^5^ A W^−1^	2.9 × 10^13^ jones at 532 nm	N/A	1.3 (532 nm)	42
	43.6−76.3 μA W^−1^ (UV-Vis)	N/A	0.2 s	N/A	229
β-GeSe_2_	N/A	N/A	N/A	3.4 (450 nm)	56
	2.5 A W^−1^ at 450 nm	1.85 × 10^8^ jones at 450 nm	0.2 s	N/A	90
GeP	3.11 A W^−1^ at 532 nm	N/A	N/A	1.83 (532 nm)	52
GeAs	6 A W^−1^ at 1.6 μm	N/A	2.5 ms	N/A	
	N/A	N/A	N/A	1.49 (520 nm) 4.4 (830 nm)	170
o-SiP	0.05 mA W^−1^ at 671 nm	N/A	0.5 s	3.14 (671 nm)	44
GeAs_2_	N/A	N/A	N/A	≈2 (532 nm)	45
GaTe	274.3 A W^−1^	4 × 10^12^ jones at 254 nm	48 ms	N/A	19
	10^4^ A W^−1^ at 532 nm	N/A	6 ms	N/A	230
	0.2 A W^−1^	N/A	2 s	N/A	231
TlSe	1.48 A W^−1^ at 633 nm	N/A	N/A	2.65	61
SnS	365 A W^−1^ at 808 nm	2.72 × 10^9^ jones at 808 nm	035 s	1.49	82
	300 A W^−1^ at 800 nm	6 × 10^9^ jones at 800 nm	36 ms	N/A	232
	156 A W^−1^ at 405 nm	2.94 × 10^10^ jones at 405 nm	5.1 ms	N/A	
	2040 A W^−1^	6 × 10^9^ jones at 800 nm	90 ms	N/A	
SnSe	N/A	N/A	N/A	2.15 at 532 nm	84
	5.5 A W^−1^ at 370 nm	6 × 10^10^ jones at 370 nm	N/A	N/A	233
	330 A W^−1^ at white light	N/A	N/A	N/A	234
TiS_3_	2910 A W^−1^	N/A	4 ms	N/A	235
	2500 A W^−1^ at 808 nm	N/A	N/A	≈4.0 (830 nm)	169
				≈4.6 (638 nm)	
				≈2.8 (532 nm)	
	5.22 × 10^2^ A W^−1^ at 1064 nm	1.69 × 10^9^ jones	1.53 s	N/A	236
Sb_2_Se_3_	1.58 A W^−1^ at 633 nm	N/A	N/A	15.05 at (633 nm)	43
ReS_2_	4 A W^−1^ at 633 nm	N/A	20 μs (633 nm)	N/A	
	1000 A W^−1^ at 532 nm	N/A	2 s (532 nm)	≈4 (532 nm)	32
	604 A W^−1^ at 500 nm	4.44 × 10^10^ jones	2 ms	N/A	237
	8.86 × 10^4^ A W^−1^ at 532 nm	1.182 × 10^12^ jones	100 s	N/A	238
	2.5 × 10^7^ A W^−1^ at 405 nm	N/A	0.67 s	N/A	239
ReSe_2_	0.1 A W^−1^ at 633 nm	N/A	2 ms	≈2	106
	2.98 A W^−1^	N/A	5.47 s	N/A	240
	95 A W^−1^ at 633 nm	N/A	68 ms	N/A	58
Ta_2_NiSe_5_	17.2 A W^−1^ at 808 nm	N/A	3.0 s	N/A	241
Td-MoTe_2_	0.4 mA W^−1^ at 532 nm	1.07 × 10^8^ jones at 532 nm	42.5 μs (532 nm) 35.8 μs (4 μm), 31.7 μs (10.6 μm)	2.72 (10.6 μm) 1.92 (4 μm), 1.19 (633 nm)	47
	4.15 × 10^−2^ mA W^−1^ at 10.6 μm	9.1 × 10^6^ jones at 10.6 μm			
Td-WTe_2_	58 A W^−1^ at 3.8 μm	N/A	0.018 s (514.5 nm), 0.85 s (3.8 μm), 11.7 s (10.6 μm)	4.9 (514.5 nm)	48
Td-TaIrTe_4_	0.34 mA W^−1^ at 633 nm	2.7 × 10^7^ jones at 633 nm	27 μs	1.13 (633 nm) 1.56 (4 μm), 1.88 (10.6 μm)	242

### 5.3 Anisotropic thermal conductivity and thermoelectric properties

Thermoelectric (TE) devices can convert heat flow into electrical energy by utilizing the Seebeck effect and the efficiency of thermoelectric conversion is described by the figure of merit,9
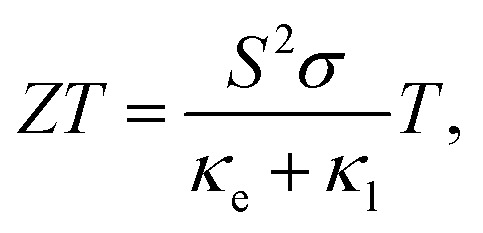
where *S*, *T*, *σ*, *κ*_e_, and *κ*_l_ are Seebeck coefficient, absolute temperature, electrical conductivity, electronic thermal conductivity, and lattice thermal conductivity, respectively. From the definition of *ZT*, we can deduce that larger *S* and *σ* with lower thermal conductivity *κ* = *κ*_e_ + *κ*_l_ should be simultaneously needed if we want to produce a TE device with outstanding performance.

Nowadays, researchers have paid great attention to the investigation of *κ* of 2D layered materials as well as their anisotropic properties. *κ* can be measured using micro-Raman method, micro-bridge method, time domain thermo-reflectance (TDTR), and time-resolved magneto-optical Kerr effect (TR-MOKE). In micro-Raman measurements, the suspending samples are transferred onto the micro-fabricated trenches or holes. The laser heats up the samples and creates a temperature gradient in the samples. Meanwhile, by measuring the Raman peak shift with temperature, we can obtain the in-plane thermal conductivity with the help of laser absorption and geometry.^171,172^ The micro-bridge method was originally used to measure the thermal conductivity of one-dimensional (1D) nanotubes or nanowires.^173^ Recently, this method has been developed to detect the thermal conductivity of 2D materials.^174^ The samples are transferred on the two suspended micro-fabricated silicon dioxide membrane islands several microns apart. One of them is the heating membrane and the other one is the sensing membrane. There are two metal resistors under the two suspended islands and a direct current is applied to the metal resistors. Consequently, the current gives rise to a temperature gradient in the sample owing to Joule heating effect. The temperature can be extracted from the resistance change of the sample and thus, we can calculate the thermal conductivity of the sample. The principle of TDTR method is to measure the thermo-reflectance response as a function of delay time between the arrival of the pump and probe pulses on the sample surface. The modulation of the pump beam at rf frequencies is used for lock-in detection of the thermoreflectance signal and to generate useful heat accumulation effects. The in-phase signal from the lock-in outputs *V*_in_ is approximately proportional to the temperature difference induced from pump pulse and the out-of-phase signal *V*_out_ is approximately proportional to the imaginary part of the temperature oscillations of the pump beam. We calculate the ratio between *V*_in_ and *V*_out_ voltages,

10 *R* = −*V*_in_/*V*_out_,

and fit them to a heat transfer model, from which the unknown thermal conductivities can be obtained. The experimental setup of TR-MOKE method is quite similar to TDTR. TR-MOKE method is used to detect the temperature dependence of magnetization through the rotation of polarization of the reflected light resulting from the MOKE.

**Fig. 16:**
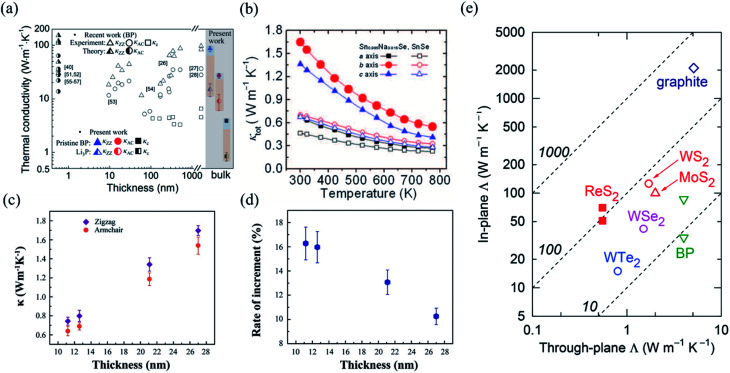
(a) Summarization of thermal conductivity measurement in three different directions plotted in comparison to the reported values as a function of thickness. Thermal conductivities of black phosphorus for the zigzag (blue), the armchair (red), and cross-plane (black) are shown to illustrate the thermal regulation range from the fully charged state (filled symbols) to the fully discharged state (half-filled symbols). Reproduced from ref. 178 with permission from American Chemical Society. (b) Total thermal conductivity as a function of temperature for SnSe crystals. Reproduced from ref. 14 with permission from American Association for the Advancement of Science. (c) The extracted thermal conductivities along the zigzag and armchair directions of Td-WTe_2_ with different thicknesses. (d) The anisotropic difference in thermal conductivities as a function of sample thickness. Reproduced from ref. 181 with permission from Wiley-VCH. (e) Thermal conductivity of 2D materials whose in-plane and through-plane thermal conductivities in the bulk limit have been experimentally determined at room temperature. The data are taken from graphite,^184^ MoS_2_,^183^ WS_2_,^185^ WSe_2_,^186^ WTe_2_,^187^ and BP.^177^ The dashed lines represent constant anisotropy ratio (*i.e.*, the in-plane to through-plane thermal conductivity ratio).^182^ Reproduced from ref. 182 with permission from Wiley-VCH.

Recently, many groups have investigated the thermoelectric behaviors of BP. Since the puckered crystal structure of BP results in a lower lattice anharmonicity and larger group velocity along the zigzag direction than the armchair direction, the thermal conductivity (*κ*) along the armchair direction is several times smaller than that along the zigzag direction, as summarized by Kang *et al.* in Fig. 16(a).^175–178^ The difference in the measured in-plane anisotropic thermal conductivity may be related to the different measuring methods and the easily degenerated surface of BP.^175,179,180^ As seen in Fig. 16(a), the 3D anisotropic thermal conductivities also have thickness dependence in the specific region, which indicates the efficiency of surface or boundary scattering.^175^ More results have proved that when the vibrations or the propagation directions of phonons are out-of-plane, the scattering is strongly enhanced, which results in the lowest thermal conductivity. But when vibrations and propagation directions of the phonons are in-plane (zigzag or armchair axis), the phonon relaxation time is almost the same. Thus, the phonon relaxation time only contributes to anisotropy in the through-plane thermal conductivity but not the in-plane thermal conductivity.^176,180^

Kang *et al.* have also developed a method to reversibly modify the thermal conductivity of BP by Li ion intercalation. The thermal conductivities of pristine BP and Li_3_P are found to be highly anisotropic, as shown in Fig. 16(a), which shows that Li ion intercalation covers a remarkably large thermal conductivity tuning range.^178^ Recently, Zhao *et al.* have reported that the *ZT* values of SnSe crystal are extremely high owing to its ultralow lattice thermal conductivity for the distinctive anharmonic structure of SnSe.^12,14^ Similar to BP, SnSe also has in-plane anisotropic *ZT* values (2.6 and 2.3 at 950 K along the *b* and *c* axes, respectively) and thermal conductivities along different axes as shown in Fig. 16(b).^14,178^ Strikingly, when SnSe is hole doped with Na, the values of thermal conductivities along three directions are enhanced due to the multiple valence band maxima that lie close together in energy by lifting the Fermi level deep into the band structure. Chen *et al.* have explored the in-plane anisotropic thermal conductivity of Td-WTe_2_ flakes with different thickness using micro-Raman spectroscopy method.^181^ The extracted thermal conductivity of the WTe_2_ samples with different thickness are shown in Fig. 16(c). Especially for the 11.2 nm thick few-layered WTe_2_, the thermal conductivity along the zigzag direction, *κ*_zigzag_ = 0.743 W m^−1^ K^−1^, is 16.3% larger than that along the armchair direction, *κ*_armchair_ = 0.639 W m^−1^ K^−1^, thus showing a strong anisotropy in the thermal conductivity. But as the thickness of WTe_2_ increases, the anisotropy of the in-plane thermal conductivity decreases due to the rise of mean free path along the armchair and less phonon-boundary scattering, as shown in Fig. 16(d).

As a typical low-symmetry 2D material, ReS_2_ also has anisotropic thermal conductivity, which has been studied using the TDTR method by Jang *et al.*^182^ They found that the thermal conductivity along the Re-chains was larger than that along the direction transverse to the chains. Apart from that, owing to the weak interlayer coupling in ReS_2_, the through-plane thermal conductivity is very low (0.55 ± 0.07) W m^−1^ K^−1^ compared with other 2D materials such as MoS_2_ and BP.^177,183^Fig. 16(e) plots a summary of 2D materials whose in-plane and through-plane thermal conductivities have been experimentally measured in the bulk limit at room temperature. From Fig. 16(e), we can see that the thermal conductivity of ReS_2_ has a remarkably high anisotropy (130 ± 40 and 90 ± 30) for the two in-plane directions.

It is well known that heavy elements are preferred for thermo-electrical devices with high performance due to the enhanced phonon scattering and lower thermal conductivity.^188^ Therefore, it has been predicted and experimentally demonstrated that Te is a good candidate as a thermoelectric material due to its high electrical conductivity and low thermal conductivity.^69,70,74^ Peide D. Ye *et al.* recently fabricated a state-of-the-art thermoelectric device based on few-layered 2D Te flakes.^15^ The Seebeck coefficient of few-layered Te can be found to be 413 μV K^−1^. Then, the thermal conductivity along the 1D chain direction of a similar suspended 2D Te flake is measured by micro-Raman spectroscopy and can be obtained to be about 1.50 W m^−1^ K^−1^. Hence, the calculated value of *ZT* for few-layered Te is about 0.63 at *T* = 300 K.

Many groups have theoretically predicted that BP has excellent thermoelectric properties.^189–191^ For example, Zhang *et al.* calculated and concluded the peak *ZT* of 1.1 and 0.6 with high electron and hole doping at 800 K.^190^ However, few reports have been aimed at investigating the TE properties of BP in experiment. Yu Saito *et al.* have currently measured and successfully tuned the Seebeck coefficient of multilayered BP by gate voltage.^192^ The maximum of *S* can reach as high as 510 μV K^−1^ at 210 K when BP is in the hole-depleted region, which is much higher than the reported bulk single crystal value of 340 μV K^−1^ at 300 K.^189^ Zhao *et al.* have previously proved that single crystals of p-type SnSe exhibited an extremely high *ZT* of ∼2.6 at 923 K along crystallographic *b*-direction.^12^ Lately, n-type Br-doped SnSe single crystals have exhibited a striking *ZT* of 2.8.^193^ Other low-symmetric 2D materials with low thermal conductivities and highly anisotropic transport properties also show potential promising thermoelectric applications. Tasuku Sakuma *et al.* recently measured the thermoelectric power *S* = −530 μV K^−1^ and calculated *ZT* = 0.0023 for quasi-one-dimensional TiS_3_ microribbon.^194^

### 5.4 Ferroelectric and piezoelectric properties

Realizing ferroelectricity and piezoelectricity in 2D layered materials is intriguing for fundamental science and potential applications (*e.g.*, non-volatile memories, generators, and sensors). Ferroelectricity is a characteristic of certain materials that have a spontaneous electric polarization that can be reversed by an external electric field. Up to now, ferroelectricity has been successfully detected in monolayer SnTe, few-layered α-In_2_Se_3_, and CuInP_2_S_6_ flakes by different methods of measurement.^195–199^ The ferroelectric behavior usually originates from the breaking of the structural centrosymmetry in the polarization direction. However, there are few reports on achieving ferroelectricity in low-symmetry layered materials, which is extremely impressionable to strain tuning with novel anisotropic properties. Recently, Fei *et al.* revealed that the unique ionic-potential anharmonicity can induce spontaneous in-plane ferroelectricity in monolayer group-IV monochalcogenides MX (M = Ge, Sn; X = S, Se). They deduced that the ferroelectricity in these materials was robust and their Curie temperatures are all above 300 K. The spontaneous electric polarization is in the order of 10^−10^ C m^−1^.^200^ The monolayer β-GeSe with puckered lattice structure was also predicted to be a 2D ferroelectric material by Guan *et al.* The in-plane spontaneous electric polarization is about 0.16 nC m^−1^, which is comparable to that of monolayer SnTe. The intrinsic Curie temperature *T*_c_ is calculated to be 212 K by using Monte Carlo simulations.^201^

Fei *et al.* have also found that two- or three-layer metallic Td-WTe_2_ exhibits spontaneous out-of-plane electric polarization that can be switched by gate in experiment.^202^ The authors estimated that the polarization intensity was about 2 × 10^−4^ C m^−2^, which was about three orders of magnitude lower than that of classic ferroelectric BaTiO_3_.^203^ Moreover, the researchers also found that the ferroelectric switching characteristics can be effectively tuned by the gate bias. The above observations are practical for ferroelectric applications and may be relevant to novel physical phenomena such as extreme and anisotropic magnetoresistance,^37,155^ a polar axis, and Weyl points.^204,205^

Since the ion-displacement of compounds can induce the dipole moment, most of the reported 2D ferroelectric materials are compounds. In comparison, elemental materials are predicted to have no ferroelectricity because there is no electronegativity difference in them. However, Wang *et al.* recently predicted that 2D few-layered tellurium is a stable ferroelectric material at temperature up to 600 K and the in-plane electric polarization is about 0.16 nC m^−1^.^206^ The origin of polarization is the in-plane ion-displacement due to interlayer interactions between the lone-pairs.

Piezoelectric effect is the electric charge accumulated in the material in response to applied mechanical stress and has been used in several devices such as piezoelectric-gated diodes, field effect transistors, and nano-sensors.^207^ Electric polarization is caused by broken symmetry and exists in most of the non-centrosymmetric materials such as ZnO and Pb(Zr_*x*_Ti_1−*x*_)O_3_ (PZT).^208^ Recently, two dimensional piezoelectric materials have attracted tremendous interest because of their good ability to endure enormous strain. It should be noted that there are some materials in which the inversion symmetry can be preserved in the 3D forms but broken in the 2D ones.^209,210^ For example, bulk MoS_2_ is not piezoelectric but Wang *et al.* have proved that the monolayer MoS_2_ flake can produce a piezoelectric voltage of 15 mV and a current of 20 pA with 0.53% strain.^102^ For the low-symmetry 2D materials, Fei *et al.* have predicted that there exists giant anisotropic piezoelectric effects in monolayer group-IV monochalcogenides. By virtue of their unique puckered *C*_2v_ symmetry and electronic structure, the piezoelectric coefficients of the monolayer group-IV monochalcogenides are surprisingly one to two orders of magnitude larger than other 2D piezoelectric materials such as MoS_2_, hexagonal BN (h-BN), and GaSe.^209,211–213^ However, as far as we know, no experimental results have been reported for measuring the piezoelectric voltage or current in monolayer group-IV monochalcogenides. The attempt of exploring anisotropic piezoelectric polarization in low-symmetry 2D materials may offer new possibilities to tailor in-plane anisotropic piezoelectric response in nanotechnology and new accesses for harvesting of energy, which can be further used for self-running nano-devices without using additional energy.

## 6. Applications

**Fig. 17:**
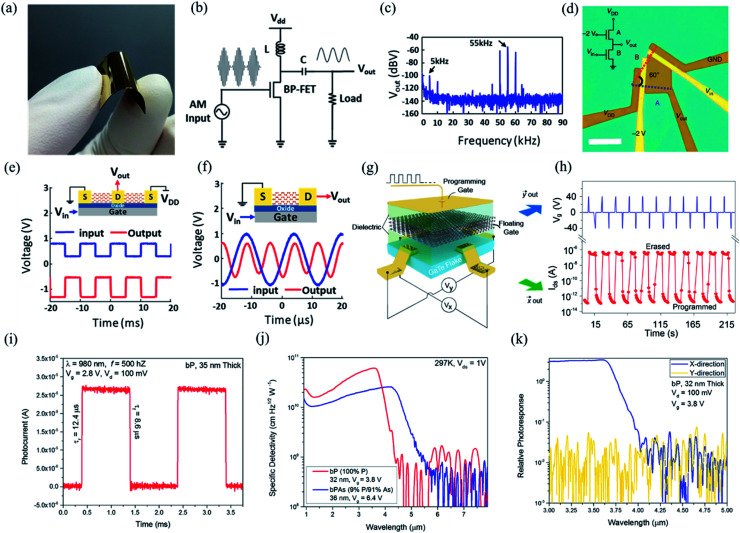
(a) Optical image of few-layer BP devices on a flexible substrate. (b) Schematic of an ideal AM demodulator system based on BP FET operating at the ambipolar point. (c) Output spectrum of the ambipolar transistor demodulator showing the demodulated baseband signal (5 kHz) and the modulated carrier feed-through (55 kHz). Input carrier *V*_pp_ = 100 mV. Reproduced from ref. 214 with permission from American Chemical Society. (d) Optical image of a typical inverter device based on few-layer ReS_2_ flake. The scale bar is 10 mm. The inset is the circuit diagram of the inverter, where the top-gate voltage along the *a*-axis is fixed at 2 V, the top-gate voltage along the *b*-axis is the input voltage *V*_in_, and the middle shared electrode is the output voltage *V*_out_. Reproduced from ref. 35 with permission from Nature Publishing Group. (e) The ambipolar digital inverter based on BP at *V*_g_ = −0.46 V and *V*_dd_ = −2 V. Input pulse oscillates at 100 Hz with peak-to-peak amplitude (*V*_pp_) of 0.5 V. Inverter output pulse showing a gain of about 1.6. (f) Ambipolar single FET frequency doubler based on BP at *V*_g_ = 0 V and *V*_d_ = −1 V. Input signal is 64 kHz sinusoid with *V*_pp_ = 2 V. Output signal oscillates at the double frequency (128 kHz) with a voltage gain of about 0.7. Reproduced from ref. 214 with permission from American Chemical Society. (g) The schematic view of a typical anisotropic memristor based on h-BN/GaTe/h-BN heterojunction with a graphite floating gate. (h) Demonstration of erasing and programming pulses measured in the *y*-direction. Reproduced from ref. 36 with permission from Nature Publishing Group. (i) Room-temperature temporal photoresponse of a 35 nm-thick BP photoconductor, excited by a 980 nm laser modulated at 50 kHz. (j) Specific detectivity of BP and b-PAs photoconductors with optimized thickness and gate voltage at room temperature.^217^ (k) Relative response of a BP photoconductor measured for incident light polarized along the *x* (armchair) and *y* (zigzag) directions of the BP crystal. Reproduced from ref. 217 with permission from American Chemical Society.

By taking advantage of multifunctionality of low-symmetry 2D layered materials, applications with anisotropic properties can be manufactured. Owing to the ambipolar functionality and high-mobility of BP, Zhu *et al.* have demonstrated high performance flexible amplitude-modulated (AM) demodulator, ambipolar digital inverter, and frequency doubler.^214^ The BP AM demodulator is a single-transistor circuit, which can convert RF signal to audio signal. The optical image and schematic of flexible BP AM demodulator are shown in Fig. 17(a) and (b). The FFT output signal spectrum for 100 mV input peak-to-peak carrier amplitude at 100% modulation index is shown in Fig. 17(c) and the authors further demonstrated that the BP AM demodulator worked well. Besides, BP ambipolar digital inverters and a frequency doubler are successfully manufactured based on the ambipolar transport characteristics and high drain current modulation. As shown in Fig. 17(e), a BP push–pull amplifier was fabricated. Two identical bottom gate transistors share the same drain as the output and the bottom gate as the input. The amplified inverted signal with an output/input voltage gain of ∼1.68 can be observed in Fig. 17(e). The frequency doubler is also widely used in analog circuits due to the low energy consumption. Compared to graphene, BP can offer lower power and higher power efficiency because of its lower DC power dissipation and off current. As shown in Fig. 17(f), the BP transistor was biased to realize symmetric transfer characteristic near the minimum conduction point and the output sinusoidal frequency was doubled with a conversion gain of 0.72. Similarly, Liu *et al.* have also fabricated a digital inverter based on the integration of two separated ReS_2_ FETs along two orientations, as shown in Fig. 17(d).^35^ The gain of the inverter is defined as |d*V*_out_/d*V*_in_| and can reach as high as 4.4 when *V*_DD_ = 3 V, which is comparable to the MoS_2_ inverters.^215,216^

Because of the gate-tunable anisotropic resistance in few-layered GaTe, Wang *et al.* have manufactured a prototype anisotropic memristor based on GaTe flakes with few-layered graphene as the floating gate.^36^ The schematic view of the device is shown in Fig. 17(g). Since the anisotropic transport characteristics vary largely in GaTe, the hysteresis memory curves along the *x*- and *y*-directions differ a lot, which show a clear window of memory. When the programming gate sweeps from 0 V to −20 V, the device is in the ‘off’ state along both the *x*- and *y*-directions. When the erasing gate sweeps from 0 V to 20 V, the device stays in two different ‘on’ states along the *x*- and *y*-directions. Therefore, the anisotropic memristor is realized by erasing and programming pulses measured in the *y*-direction, as shown in Fig. 17(h) and has great potential in direction-sensitive data storage.

**Fig. 18:**
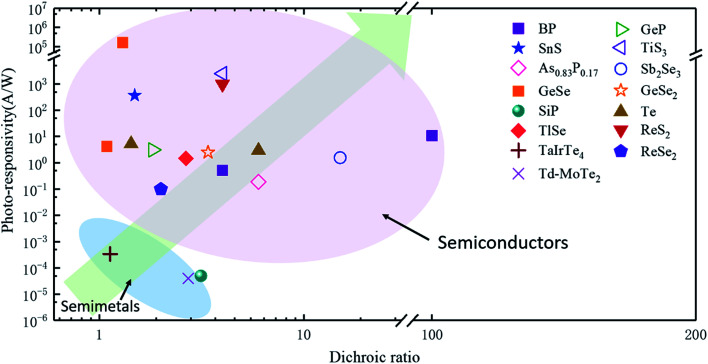
Summarization of the values of photo-responsivity and dichroic ratios for low-symmetry 2D materials. The data are extracted from Table 2.

Optoelectronic devices rely on light–matter interactions and can convert light into electrical signal or *vice versa*.^218,219^ Optoelectronic devices including detectors, lasers LEDs, solar cells, and optical switches are widely used in low-loss optical fiber communications, power generation, and military measure systems. For low dimensional and flexible photodetectors, 2D layered materials should exhibit high responsivity, large detectivity, and fast response time.^111,220–222^ Unlike graphene, many other 2D layered materials have a band gap and large absorption coefficient, which are beneficent for high performance photodetectors. In addition, polarization-sensitive photodetectors based on low-symmetry layered materials are also desirable in optical communication, remote sensing, and optical data storage.^166,167^ Apart from the photodetectors based on low-symmetry materials mentioned above, similar polarization-sensitive photodetectors have also been investigated, including wide-band-gap ultraviolet photodetectors (*e.g.*, GeS_2_), visible-light photodetectors (*e.g.*, ReS_2_, ReSe_2_, SnSe, SnS, GeSe, GeS_2_, GeSe_2_, GeP, SiP, TiS_3_, and Sb_2_Se_3_), and narrow-band-gap infrared photodetector (*e.g.*, Td-MoTe_2_, Td-WTe_2_, and TaIrTe_4_). For comparison, more performance details of a series of 2D-based polarization-sensitive photodetectors are listed in Table 2. Take the high-performance polarization-sensitive photodetectors based on BP for example.^217^ As shown in Fig. 17(i) and (j), the photo-response time is ultrafast, which is measured to be about 12.4 μs. The specific detectivity (D*) can be optimized by adjusting the thickness of BP to maximize the absorption and minimize the dark current. The maximum value of D* can reach as high as 6 × 10^10^ jones at room temperature, which is about one order of magnitude higher than commercial mid-wave infrared detectors operating at room temperature. As another critical index, the dichroic ratio of photocurrent can be obtained in Fig. 17(k). One can find that the polarization ratio (dichroic ratio) between the two crystal orientations of BP at mid-infrared wavelengths is larger than 100, which is larger than all the other low-symmetry 2D materials. This value is limited by experimental instruments and approaches the extinction ratio of the polarizer used in this study. Since both photo-responsivity and dichroic ratio are the most important indices for the polarization-sensitive photodetectors, here, we have summarized these two values of some low-symmetry 2D materials. As we can see from Fig. 18, both photo-responsivity and dichroic ratios should be high for extraordinary polarization-sensitive photodetectors, which provides guidance for next-generation promising optoelectronic devices with in-plane anisotropy.

## 7. Conclusions and outlook

Here, we have summarized the recent achievements in low-symmetry 2D layered materials and their anisotropic properties, including anisotropic electronic, optoelectronic, magnetic transport, thermoelectric, piezoelectric, and ferroelectric properties, resulting from their anisotropic structures and band structures. On account of the intriguing anisotropic electronic properties, the applications have been fabricated and developed, such as in-plane anisotropic FETs,^63,243^ anisotropic floating gate memristors,^36^ digital inverters,^35,244^ memristors, and polarization-sensitive photodetectors.^31,32,45,53,56,228^

However, there are still many problems to be resolved to attain a comprehensive understanding of the properties of low-symmetry 2D materials and to realize their full potential in multifunctional fields. The potential opportunities and challenges are listed as follows: (1) more work is needed to achieve low-symmetry 2D materials at a large scale. Although a few low-symmetry 2D materials (*e.g.*, SnS and GeSe) have already been manufactured by CVD and PVD methods, still a lot of few-layered low-symmetry 2D materials have only been made by exfoliation, which limits the development of fabrication devices with anisotropic properties. (2) Since the anisotropic ratio of anisotropic 2D materials is still very low (for most of them, it is less than three), exploring new materials and techniques to enhance the in-plane anisotropy of 2D materials is very essential and promising for future anisotropic devices. (3) Searching new methods to effectively modulate and enhance the in-plane anisotropic ratio. Recently, Wang *et al.* have discovered that the in-plane anisotropic ratio of resistance in few-layered GaTe can be greatly enlarged by tuning the gate voltage.^36^ But for other low-symmetry 2D materials, whether the gate voltage can also modulate the in-plane anisotropic properties is still unknown. (4) The predicted thermoelectric, piezoelectric, and ferroelectric properties and their anisotropy in some of the low-symmetry 2D materials are still needed to be confirmed and explored by experiments. (5) Although many researchers have deeply investigated the properties of heterostructures based on low-symmetry 2D materials, the isotropic/anisotropic and anisotropic/anisotropic 2D stacked heterostructures require more in-depth study to elucidate the unique properties and upgrade the device performance. (6) Since Wu *et al.* have predicted that moiré bands of twisted transition metal dichalcogenide homo-bilayers can be topologically non-trivial,^245^ new physical properties such as quantum spin Hall effect and superconductivity may be observed in twisted bilayer of some particular low-symmetry 2D materials. Overall, the recent findings concerning anisotropic electronics indicate a broad promise in multifunctional applications.

## Conflicts of interest

The authors declare no competing financial interests.

## Supplementary Material
